# Patterns of synchronization in 2D networks of inhibitory neurons

**DOI:** 10.3389/fncom.2022.903883

**Published:** 2022-08-16

**Authors:** Jennifer Miller, Hwayeon Ryu, Xueying Wang, Victoria Booth, Sue Ann Campbell

**Affiliations:** ^1^Mathematics Department, Bellarmine University, Louisville, KY, United States; ^2^Department of Mathematics and Statistics, Elon University, Elon, NC, United States; ^3^Department of Mathematics and Statistics, Washington State University, Pullman, WA, United States; ^4^Departments of Mathematics and Anesthesiology, University of Michigan, Ann Arbor, MI, United States; ^5^Department of Applied Mathematics and Centre for Theoretical Neuroscience, University of Waterloo, Waterloo, ON, Canada

**Keywords:** inhibitory networks, synchronization, phase models, stability analysis, cluster solutions, 2D networks, block circulant matrices, connectivity

## Abstract

Neural firing in many inhibitory networks displays synchronous assembly or clustered firing, in which subsets of neurons fire synchronously, and these subsets may vary with different inputs to, or states of, the network. Most prior analytical and computational modeling of such networks has focused on 1D networks or 2D networks with symmetry (often circular symmetry). Here, we consider a 2D discrete network model on a general torus, where neurons are coupled to two or more nearest neighbors in three directions (horizontal, vertical, and diagonal), and allow different coupling strengths in all directions. Using phase model analysis, we establish conditions for the stability of different patterns of clustered firing behavior in the network. We then apply our results to study how variation of network connectivity and the presence of heterogeneous coupling strengths influence which patterns are stable. We confirm and supplement our results with numerical simulations of biophysical inhibitory neural network models. Our work shows that 2D networks may exhibit clustered firing behavior that cannot be predicted as a simple generalization of a 1D network, and that heterogeneity of coupling can be an important factor in determining which patterns are stable.

## 1. Introduction

In different brain regions, neural firing patterns can be characterized as coordinated assembly activity, in which individual collections of cells fire synchronously and not at the same time as other collections of synchronously firing cells (Gray et al., 1989; Laurent and Davidowitz, 1994; Harris et al., 2003; Dragoi and Buzsáki, 2006; Muldoon et al., 2013; Barbera et al., 2016). Neural assemblies may consist of spatially localized neurons (Muldoon et al., 2013; Barbera et al., 2016) or of neurons that are widespread across one or more brain regions (Gray et al., 1989; Harris et al., 2003). For example, neural assemblies have been observed between neurons in different cortical columns (Gray et al., 1989), within regions of the hippocampus (Harris et al., 2003; Dragoi and Buzsáki, 2006), the dentate gyrus (Muldoon et al., 2013), and between cells in the striatum (Carrillo-Reid et al., 2008; Adler et al., 2013; Barbera et al., 2016) and the olfactory bulb (Laurent and Davidowitz, 1994). Understanding the dynamics and formation of synchronized assemblies within larger neural networks has gained increasing importance in neuroscience (Engel et al., 2001) and has been studied both experimentally (Laurent and Davidowitz, 1994; Harris et al., 2003; Galán et al., 2006; Muldoon et al., 2013; Barbera et al., 2016) and using computational modeling (Golomb et al., 1992; Golomb and Rinzel, 1994; Li et al., 2003; Achuthan and Canavier, 2009; Canavier et al., 2009; Ponzi and Wickens, 2010; Kilpatrick and Ermentrout, 2011; Angulo-Garcia et al., 2016).

One approach to understanding the formation of neural assemblies is through the study of cluster solutions in model networks of intrinsically oscillating neurons (Golomb et al., 1992; Golomb and Rinzel, 1994; Li et al., 2003; Galán et al., 2006; Kilpatrick and Ermentrout, 2011; Miller et al., 2015; Campbell and Wang, 2018; Ryu et al., 2021). Cluster solutions are solutions where the network of oscillators breaks into subgroups. Within each subgroup, the phases of the oscillators are the same, while oscillators in different subgroups are phase-locked with some non-zero phase difference. A useful mathematical framework for studying cluster solutions is the phase model reduction (Hoppensteadt and Izhikevich, 1997; Schwemmer and Lewis, 2012). This framework has been used to study synchronization and clustering in a variety of coupled oscillator networks (Ashwin and Swift, 1992; Okuda, 1993; Kopell and Ermentrout, 2002; Saraga et al., 2006; Mancilla et al., 2007). A complementary approach is to consider a continuum model representing the limit of an infinite number of oscillators (Ermentrout, 1992; Strogatz, 2000). In such models, which are represented as partial differential equations, cluster solutions correspond to wave-like solutions, sometimes called twisted states (Ermentrout, 1992; Wiley et al., 2006; Kazanci and Ermentrout, 2007; Girnyk et al., 2012; Heitmann et al., 2012; Heitmann and Ermentrout, 2015).

Cluster solutions have been extensively studied in models that assume homogeneous, all-to-all connectivity among oscillators (Sakaguchi and Kuramoto, 1986; Ashwin and Swift, 1992; Hansel et al., 1993; Okuda, 1993; Strogatz, 2000; Zanette, 2000; Kazanci and Ermentrout, 2008; Campbell and Wang, 2018). The number of studies considering cluster solutions in networks with structured connectivity is more limited and they primarily focus on networks with neurons arranged in a 1D ring, of arbitrary size *N*. In Kazanci and Ermentrout (2007, 2008), the phase model reduction is used to analyze how the addition of local (nearest neighbor) gap-junctional coupling in a network with all-to-all synaptic coupling affects cluster solutions. They found that gap-junctional coupling can induce a shift from synchronous or two cluster solutions to an *N*-cluster solution. The presence of time-delayed synaptic connections among all-to-all coupled oscillators was analyzed using a more general phase model in Campbell and Wang (2018). They found that time delays can lead to the coexistence of multiple stable clustering solutions. In a continuum network model, the existence and stability of cluster solutions when each oscillator has identical coupling to a subset of its nearest neighbors was analyzed in Wiley et al. (2006); Girnyk et al. (2012). In a study considering structured excitatory-inhibitory coupling in a continuum model (Heitmann and Ermentrout, 2015), the extent of inhibitory coupling was shown to influence the existence of synchronous or traveling wave solutions in both 1D and 2D networks. Recently, a phase model analysis of cluster solutions was extended to 2D networks in Culp (2021), in which the existence of cluster solutions and stability conditions of a particular type of clusters (that have identical phase difference between adjacent oscillators in the horizontal and vertical directions) were derived on a square torus.

In our previous work (Miller et al., 2015; Ryu et al., 2021), we used the phase model approach to determine existence and stability conditions for cluster solutions in 1D, discrete inhibitory neural networks with various connectivity schemes. In Miller et al. (2015) we investigated the clustering dynamics of a network of oscillating inhibitory neurons in which each neuron is coupled to its two nearest neighbors on each side. Our phase model analysis showed that changing the connection weights can change the stability of solutions, confirmed by numerical simulations. In Ryu et al. (2021), we considered simple non-monotonic, distance-dependent connectivity schemes in 1D inhibitory networks. We similarly used the phase model approach to identify conditions for the existence and stability of cluster firing solutions in which clusters consist of spatially adjacent neurons in inhibitory neural networks.

In this paper, we extend our previous work to a 2D, discrete inhibitory network model on a general torus, where neurons are coupled to two or more nearest neighbors in three directions (horizontal, vertical, and diagonal) with different coupling strengths in all directions. Using phase model analysis, we derive stability conditions for different patterns of clustered firing behavior in the networks, and further explore the effect of heterogeneous coupling strengths on the stability of cluster solutions. Stability conditions are derived for a general phase model, but applied to the specific phase model derived from a biophysical network model consisting of inhibitory interneurons (Wang and Buzsáki, 1996). We show that the phase model predictions give an accurate picture of the effect of various connectivity schemes on the solutions that occur in the full biophysical model.

Our paper is structured as follows: Section 2 provides the description of the methodology we employ, including the reduction from a biophysical model to a phase model (Section 2.1) and the specific biophysical neuron model used for numerical simulations (Section 2.2). Section 3 describes our analysis of the existence and stability of cluster solutions in a general phase model. Section 4 describes the application of these results to the specific phase model corresponding to the biophysical network and the verification of these results *via* numerical simulations. We conclude with a discussion of our results in Section 5.

## 2. Methods

### 2.1. Phase reduction method

In this work, we consider a general neural network model on a 2D lattice that consists of *m*×*n* identical, weakly coupled, inhibitory oscillating cells with periodic boundary conditions:


(1) $$\begin{array}{l} \frac{dX_{ij}}{dt}=F\left(X_{ij}\right)+\epsilon \underset{p,q}{\sum }w_{p,q}G\left(X_{ij},X_{i+p,j+q}\right),\\ \;0\leq i\leq n-1,0\leq j\leq m-1. \end{array}$$

Here, each *X*_*ij*_ defines a *k*-dimensional variable. *F*:ℝ^*k*^ → ℝ^*k*^ is the internal vector field of the isolated oscillator and *G*:ℝ^*k*^ × ℝ^*k*^ → ℝ^*k*^ is the synaptic coupling function. *W* = (*w*_*p, q*_) is the connection matrix with *w*_*p, q*_ > 0 when a synapse exists from cell *q* to cell *p*. We apply the phase reduction method to the above model to study (i) the existence and stability of certain cluster solutions on a 2D general torus network, and (ii) how the coupling structure on the 2D networks can affect the stability of these cluster solutions.

In the remaining part of this subsection, we will review the phase reduction method. Interested readers can refer to Hoppensteadt and Izhikevich (1997); Ermentrout and Terman (2010); Schwemmer and Lewis (2012) for more details.

Each uncoupled single neuron is assumed to admit an exponentially asymptotically stable *T*-periodic orbit, $\left\{\hat{X}\left(t\right):0\leq t\leq T=2\pi /\Omega \right\}$, and $\hat{X}\left(t\right)$ satisfies


(2) $$\begin{array}{l} \frac{dX}{dt}=F\left(X\left(t\right)\right),\;X\in {\mathbb{R}}^{k}. \end{array}$$

By the theory of weakly coupled oscillators (see Hoppensteadt and Izhikevich, 1997; Ermentrout and Terman, 2010; Schwemmer and Lewis, 2012), the complete state of each neuron in the network can be approximately captured by its phase on the standardized limit cycle θ(*t*), for 0 ≤ *t* <2π. This decreases the number of equations that describe an uncoupled single neuron from *k* equations to one, which in turn can significantly reduce the dimension of the neural network model. The phase of the *ij*^*th*^ neuronal oscillator is slowly varying and its dynamics is governed by


(3) $$\begin{array}{l} \frac{d\theta_{ij}}{dt}=\Omega +\epsilon \underset{p,q}{\sum }w_{p,q}H\left(\theta_{i+p,j+q}-\theta_{ij}\right), \end{array}$$

where


(4) $$\begin{array}{l} H\left(\psi \right)=\frac{1}{T}\int_{0}^{T}Z\left(t\right)G\left[\hat{X}\left(t\right),\hat{X}\left(t+\psi /\Omega \right)\right]dt. \end{array}$$

The function *H*, often called the interaction function, measures the modulation of the instantaneous phase of the *i*^*th*^ oscillator due to its coupling with other oscillators on the network. The function *Z* is the unique periodic solution of the linearized adjoint system


$$\begin{array}{l} \frac{dZ}{dt}=-{\left[DF\left(\hat{X}\left(t\right)\right)\right]}^{T}Z, \end{array}$$

satisfying the normalization condition


$$\begin{array}{l} \frac{1}{T}\int_{0}^{T}Z\left(t\right)\cdot F\left(\hat{X}\left(t\right)\right)dt=1. \end{array}$$

### 2.2. Neural network model

We apply our theoretical results to a biophysical inhibitory neural network model, where each individual neuron is modeled by the conductance-based Wang and Buzsáki inhibitory interneuron model (Wang and Buzsáki, 1996). Networks consist of *N* neurons coupled in a 2D lattice structure through inhibitory synaptic currents with *N* = *m*×*n*. Membrane voltage of the *i*th cell, *V*_*i*_ (in mV) is modeled by:


(5) $$\begin{array}{c} \begin{array}{l} \;C\frac{dV_{i}}{dt}=I_{app}\\ \;-g_{Na}m_{\infty }^{3}\left(V_{i}\right)h_{i}\left(V_{i}-V_{Na}\right)-g_{K}n_{i}^{4}\left(V_{i}-V_{K}\right)\\ \;-g_{L}\left(V_{i}-V_{L}\right)\\ \;-g_{syn}\left(V_{i}-V_{syn}\right)\overset{N}{\underset{j=1}{\sum }}W_{ij}s_{j},\\ \;=I_{app}-I_{ion}\left(V_{i},h_{i},n_{i}\right)-I_{syn}\left(\vec{V},\vec{s}\right)=f^{V}\left(\vec{V},h_{i},n_{i},\vec{s}\right),\\ \frac{dh_{i}}{dt}=\gamma \left(\alpha_{h}\left(V_{i}\right)\left(1-h_{i}\right)-\beta_{h}\left(V_{i}\right)h_{i}\right)=f^{h}\left(V_{i},h_{i}\right),\\ \frac{dn_{i}}{dt}=\gamma \left(\alpha_{n}\left(V_{i}\right)\left(1-n_{i}\right)-\beta_{n}\left(V_{i}\right)n_{i}\right)=f^{n}\left(V_{i},n_{i}\right),\\ \frac{ds_{i}}{dt}=-\frac{s_{i}}{\tau_{inh}}+\alpha_{inh}\left(V_{i}\right)\left(1-s_{i}\right), \end{array} \end{array}$$

with


(6) $$\begin{array}{l} \begin{array}{l} {\;m}_{\infty }^{3}\left(V\right)=\frac{-0.1\left(V+35\right)/\left(\exp\left(-0.1\left(V+35\right)\right)-1\right)}{4\exp\left(-\left(V+60\right)/18\right)},\\ {\;\alpha }_{h}\left(V\right)=0.07\exp\left(-\left(V+58\right)/20\right),\\ {\;\beta }_{h}\left(V\right)=1/\left(\exp\left(-0.1\left(V+28\right)\right)+1\right),\\ {\;\alpha }_{n}\left(V\right)=-0.01\left(V+34\right)/\left(\exp\left(-0.1\left(V+34\right)\right)-1\right),\\ {\;\beta }_{n}\left(V\right)=0.125\exp\left(-\left(V+44\right)/80\right),\\ \alpha_{inh}\left(V\right)=\alpha_{0}/\left(1+\exp\left(-V/5\right)\right), \end{array} \end{array}$$

where *h*_*i*_ is the gating variable governing inactivation of the inward sodium current, *n*_*i*_ is the gating variable governing activation of the outward potassium current and *s*_*i*_ is the gating variable for the synaptic current generated by presynaptic cell *i*. Coupling between neurons is dictated by the connectivity matrix *W*_*ij*_ and maximum synaptic strength is set to *g*_*syn*_. The definition of model parameters and their default values are provided in Table 1.

**Table 1:** Description of parameters and the values used for the neuron model.

**Parameter**	**Description**	**Value**
γ	Adjusts reaction rates for temperature	1
*g*_*Na*_	Maximal sodium conductance	35 mS/cm^2^
*g*_*K*_	Maximal potassium conductance	9 mS/cm^2^
*g*_*L*_	Maximal leak conductance	0.1 mS/cm^2^
*V*_*Na*_	Sodium reversal potential	55 mV
*V*_*K*_	Potassium reversal potential	−90 mV
*V*_*L*_	Leak reversal potential	−65 mV
*C*	Membrane capacitance	1 μF/cm^2^
*I*_*app*_	Applied current	0.4 μA/cm^2^
*V*_*syn*_	Synapse reversal potential	−75 mV
*g*_*syn*_	Maximal synaptic conductance	0.05 mS/cm^2^
α_0_	Synaptic maximal activation rate	4 ms^−1^
τ_*inh*_	Synaptic decay time	2 ms

## 3. Analysis results

**Figure 1:**
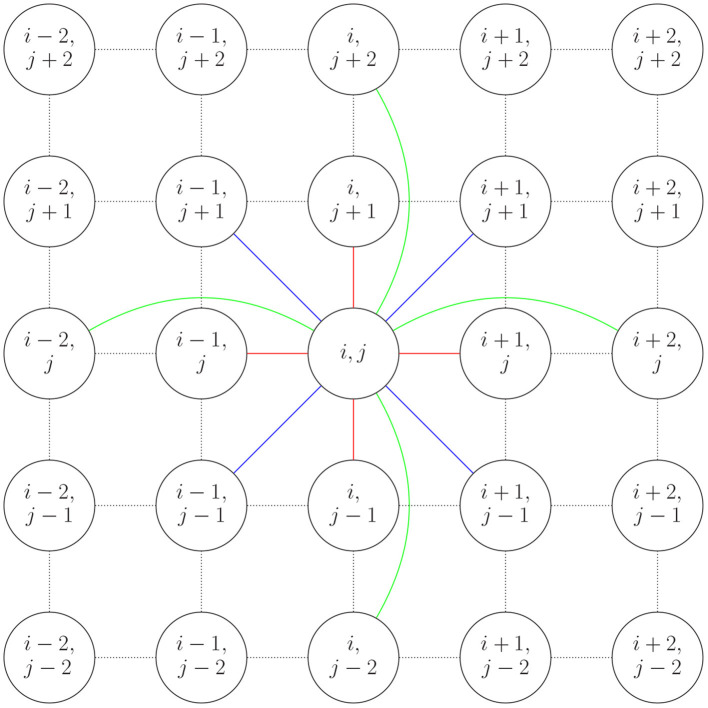
The connections to the 12 neighbors of the neuron *i, j* are shown by solid edges. Connections to nearest neighbors in the vertical and horizontal directions are shown in red. Connections to diagonal neighbors are blue. Connections to second nearest neighbors vertically and horizontally are green.

We consider the network on a perfect *m*×*n* lattice. We can define coupling in the network by letting S={(1,0), (-1,0), (0, 1), (0, -1), (-1, 1), (1,1), (1,-1), (-1, -1), (2, 0), (-2, 0), (0, 2), (0, -2)}. A network of phase oscillators with *S*-coupling means that each neuron is connected to its 8 nearest neighbors (vertical, horizontal, and diagonal directions) and 4 more distant neighbors (second nearest neighbors in the vertical and horizontal directions). An example showing the 12 neighbors of one neuron is shown in Figure 1.

The network model takes the form of (3). In the above system of equations and what follows, we assume that all the horizontal (or vertical) indices are defined under the mod operation mod *n* (or *m*).

Phase-locked solutions of a phase model such as system (3), will be solutions where all oscillators evolve with the same frequency. Thus, we look for solutions of (3) in the form


(7) $$\begin{array}{l} {\bar{\theta }}_{ij}=\left(\Omega +\epsilon \omega \right)t+\phi_{ij}. \end{array}$$

Substituting (7) into (3) we find


(8) $$\begin{array}{l} \omega =\underset{\left(p,q\right)\in S}{\sum }w_{p,q}H\phi_{\left(i+p\right),\left(j+q\right)}-\phi_{ij}. \end{array}$$

Since ω must be constant and (8) should be independent of all *i, j*, we consider a special type of solution with constant phase difference along the horizontal and vertical directions. Let ψ_*h*_ and ψ_*v*_ be the phase differences between adjacent cells in the horizontal and vertical directions, respectively. For any *p*_*h*_ that divides *n*, it follows from the periodic boundary condition that a *p*_*h*_-cluster solution along the horizontal direction implies that *p*_*h*_ψ_*h*_ = 2ℓ_*h*_π for some ℓ_*h*_ < *p*_*h*_ and gcd(ℓ_*h*_, *p*_*h*_) = 1. Similarly, in the vertical direction, for any *p*_*v*_ that divides *m*, a *p*_*v*_-cluster along the vertical direction implies that *p*_*v*_ψ_*v*_ = 2ℓ_*v*_π for some ℓ_*v*_ < *p*_*v*_ and gcd(ℓ_*v*_, *p*_*v*_) = 1. For the network as a whole this gives a *p*-cluster solution with *p* = lcm(*p*_*h*_, *p*_*v*_) (i.e., *p* is the least common multiple of *p*_*h*_ and *p*_*v*_). Hence, we have the following existence result.

** Proposition 1**. *Suppose that* *p*_*h*_ *is a factor of *n*, and *p*_*v*_ is a factor of *m*. Let *p* = lcm(*p*_*h*_, *p*_*v*_). Then a torus network of *m*×*n* neurons with *S*-coupling admits a *p*-cluster solution with constant phase differences ψ_*h*_ and ψ_*v*_ between adjacent cells in the horizontal and vertical directions, respectively. These phase differences satisfy* $\psi_{h}=\frac{2\ell_{h}\pi }{p_{h}}$ *and* $\psi_{v}=\frac{2\ell_{v}\pi }{p_{v}}$ *such that ℓ_*h*_ < *p*_*h*_ and gcd(ℓ_*h*_, *p*_*h*_) = 1, and ℓ_*v*_ < *p*_*v*_ and gcd(ℓ_*v*_, *p*_*v*_) = 1.*

We define the solutions described in Proposition 1 as (*p*_*h*_, *p*_*v*_) *p***-cluster solutions**. For such a *p*-cluster solution with phase differences ψ_*h*_ and ψ_*v*_, we have that


$$\begin{array}{l} \omega =w_{1,0}H\left(\psi_{h}\right)+w_{0,1}H\left(\psi_{v}\right)\\ \;+w_{-1,0}H\left(-\psi_{h}\right)+w_{0,-1}H\left(-\psi_{v}\right)+w_{-1,1}H\left(-\psi_{h}+\psi_{v}\right)\\ \;+w_{1,-1}H\left(\psi_{h}-\psi_{v}\right)\\ \;+w_{-1,-1}H\left(-\psi_{h}-\psi_{v}\right)+w_{1,1}H\left(\psi_{h}+\psi_{v}\right)\\ \;+w_{2,0}H\left(2\psi_{h}\right)+w_{0,2}H\left(\psi_{v}\right)\\ \;+w_{-2,0}H\left(-2\psi_{h}\right)+w_{0,-2}H\left(-2\psi_{v}\right)\\ \;=\underset{\left(p,q\right)\in S}{\sum }w_{p,q}H\left(p\psi_{h}+q\psi_{v}\right) \end{array}$$

is constant.

We now determine the stability of these (*p*_*h*_, *p*_*v*_) *p*-cluster solutions. Let $\theta_{ij}={\bar{\theta }}_{ij}+u_{ij}\left(t\right)$. Linearizing (3) about ${\bar{\theta }}_{ij}$ leads to


$$\begin{array}{l} \frac{du_{ij}}{dt}\\ =\epsilon \sum_{p,q\in S}w_{p,q}[-H^{\prime }(\theta_{\left(i+p),(j+q\right)}-\theta_{ij})u_{ij}\\ +H^{\prime }(\theta_{\left(i+p),(j+q\right)}-\theta_{ij})u_{\left(i+p),\;(j+q\right)}]. \end{array}$$

Rewriting the above equation in the matrix form, we have:


(9) $$\begin{array}{l} \frac{d\vec{u}}{dt}=\epsilon \left(-cI_{N}+Ŵ\right)\vec{u}, \end{array}$$

where


$$\begin{array}{l} \vec{u}=(u_{0,0},u_{1,0},\ldots ,u_{n-1,0},u_{0,1},u_{1,1},\ldots ,u_{n-1,1},\ldots ,\\ u_{0,m-1},u_{1,m-1},\ldots ,u_{n-1,m-1}{)}^{T},\\ {\hat{w}}_{p,q}=w_{p,q}H^{\prime }(p\psi_{h}+q\psi_{v}),n\;\\ c=\sum_{(p,q)\in S}{\hat{w}}_{p,q}, \end{array}$$

and Ŵ = bcirc(Ŵ^0^, Ŵ^1^, …, Ŵ^*m*−1^) where


$$\begin{array}{l} {\hat{W}}^{0}=\text{circ}(0,{\hat{w}}_{1,0},{\hat{w}}_{2,0},0,\ldots ,0,{\hat{w}}_{-2,0},{\hat{w}}_{-1,0}),\\ {\hat{W}}^{1}=\text{circ}({\hat{w}}_{0,1},{\hat{w}}_{1,1},0,\ldots ,0,{\hat{w}}_{-1,1}),\;{\hat{W}}^{2}={\hat{w}}_{0,2}I,\\ {\hat{W}}^{m-2}={\hat{w}}_{0,-2}I,\;\\ \;{\hat{W}}^{m-1}=\text{circ}({\hat{w}}_{0,-1},{\hat{w}}_{1,-1},0,\ldots ,0,{\hat{w}}_{-1,-1}), \end{array}$$

and all other Ŵ^*k*^ are *n*×*n* zero matrices.

Let $\rho_{n}=e^{2\pi \sqrt{-1}/n}$. The eigenvalues of the non-zero Ŵ^*k*^ are


(10) $$\begin{array}{l} \;\lambda_{j}^{{\hat{W}}^{0}}={\hat{w}}_{1,0}\rho_{n}^{j}+{\hat{w}}_{-1,0}\rho_{n}^{-j},\\ \;\lambda_{j}^{{\hat{W}}^{1}}={\hat{w}}_{0,1}+{\hat{w}}_{1,1}\rho_{n}^{j}+{\hat{w}}_{-1,1}\rho_{n}^{-j},\\ \;\lambda_{j}^{{\hat{W}}^{2}}={\hat{w}}_{0,2},\\ \lambda_{j}^{{\hat{W}}^{m-2}}={\hat{w}}_{0,-2},\\ \lambda_{j}^{{\hat{W}}^{m-1}}={\hat{w}}_{0,-1}+{\hat{w}}_{1,-1}\rho_{n}^{j}+{\hat{w}}_{-1,-1}\rho_{n}^{-j}, \end{array}$$

for 0 ≤ *j* ≤ *n*−1. Let


$$\Lambda_{\ell }=\text{diag}\left[\lambda_{0}^{{\hat{W}}^{\ell }},\lambda_{1}^{{\hat{W}}^{\ell }},\cdots ,\lambda_{n-1}^{{\hat{W}}^{\ell }}\right],\;\ell =0,1,m-1.$$

The matrix Ŵ is a block circulant matrix, and each block Ŵ^*j*^, 0 ≤ *j* ≤ *m*−1, is circulant. Let $P_{m}=\text{diag}\left(1,\rho_{m},\rho_{m}^{2},\ldots ,\rho_{m}^{m-1}\right)$ and ⊗ denote the Kronecker product of matrices. By Davis (2013), the eigenvalues of Ŵ are given by the diagonal entries of


$$\begin{array}{l} I\text{ ​​}⊗\text{​ ​}\Lambda_{0}+P_{m}⊗\Lambda_{1}+P_{m}^{2}⊗\Lambda_{2}+P_{m}^{m-2}⊗\Lambda_{m-2}\\ \;+\;P_{m}^{m-1}⊗\Lambda_{m-1}\\ \;=\text{bdiag}\left[D_{0},D_{1},D_{2},0_{m},\cdots ,0_{m},D_{m-2},D_{m-1}\right], \end{array}$$

where


$$D_{i}=\underset{\ell \in L}{\sum }\rho_{m}^{\ell }\Lambda_{\ell }$$

and *L* = {0, 1, 2, *m*−2, *m*−1}.

Thus, the eigenvalues of Ŵ are


$$\begin{array}{l} \lambda_{jk}^{\hat{W}}=\lambda_{j}^{{\hat{W}}^{0}}+\rho_{m}^{k}\lambda_{j}^{{\hat{W}}^{1}}\\ +\rho_{m}^{2k}\lambda_{j}^{{\hat{W}}^{2}}+\rho_{m}^{-2k}\lambda_{j}^{{\hat{W}}^{m-2}}+\rho_{m}^{-k}\lambda_{j}^{{\hat{W}}^{m-1}},\;\\ \;0\leq j\leq n-1,0\leq k\leq m-1, \end{array}$$

which means the eigenvalues of *J* = −*cI*_*N*_+Ŵ are


$$\begin{array}{l} \lambda_{jk}^{J}=-c\%+\lambda_{jk}^{\hat{W}}\\ +\lambda_{j}^{{\hat{W}}^{0}}+\rho_{m}^{k}\lambda_{j}^{{\hat{W}}^{1}}+\rho_{m}^{2k}\lambda_{j}^{{\hat{W}}^{2}}+\rho_{m}^{-2k}\lambda_{j}^{{\hat{W}}^{m-2}}+\rho_{m}^{-k}\lambda_{j}^{{\hat{W}}^{m-1}},\\ \;0\leq j\leq n-1,0\leq k\leq m-1. \end{array}$$

By direct calculation, we find that the eigenvalues of *J* are


$$\begin{array}{l} \lambda_{jk}^{J}\\ =-[w_{1,0}H^{\prime }(\psi_{h})(1-\rho_{n}^{j})+w_{-1,0}H^{\prime }(-\psi_{h})(1-\rho_{n}^{-j})\\ +w_{0,1}H^{\prime }(\psi_{v})(1-\rho_{m}^{k})+w_{0,-1}H^{\prime }(-\psi_{v})(1-\rho_{m}^{-k})\\ +w_{1,1}H^{\prime }(\psi_{h}+\psi_{v})(1-\rho_{n}^{j}\rho_{m}^{k})\\ +w_{-1,1}H^{\prime }(\psi_{v}-\psi_{h})(1-\rho_{n}^{-j}\rho_{m}^{k})\\ +w_{1,-1}H^{\prime }(\psi_{h}-\psi_{v})(1-\rho_{n}^{j}\rho_{m}^{-k})\\ +w_{-1,-1}H^{\prime }(-\psi_{h}-\psi_{v})(1-\rho_{n}^{-j}\rho_{m}^{-k})\\ +w_{2,0}H^{\prime }(2\psi_{h})(1-\rho_{n}^{2j})\\ +w_{-2,0}H^{\prime }(-2\psi_{h})(1-\rho_{n}^{-2j})\\ +w_{0,2}H^{\prime }(2\psi_{v})(1-\rho_{m}^{2k})+w_{0,-2}H^{\prime }(-2\psi_{v})(1-\rho_{m}^{-2k})], \end{array}$$

for 0 ≤ *j* ≤ *n*−1, 0 ≤ *k* ≤ *m*−1.

The real parts of the eigenvalues are


(11) $$\begin{array}{l} ℜ\left(\lambda_{jk}^{J}\right)\\ =-[w_{1,0}H^{\prime }\left(\psi_{h}\right)\left(1-\cos\left(\frac{2\pi j}{n}\right)\right)\\ +w_{-1,0}H^{\prime }\left(-\psi_{h}\right)\left(1-\cos\left(\frac{2\pi j}{n}\right)\right)\\ +w_{0,1}H^{\prime }\left(\psi_{v}\right)\left(1-\cos\left(\frac{2\pi k}{m}\right)\right)+w_{0,-1}H^{\prime }\left(-\psi_{v}\right)\\ \left(1-\cos\left(\frac{2\pi k}{m}\right)\right)\\ +w_{-1,1}H^{\prime }\left(\psi_{v}-\psi_{h}\right)\left(1-\cos\left(\frac{2\pi j}{n}\right)\cos\left(\frac{2\pi k}{m}\right)\right)\\ +w_{1,1}H^{\prime }\left(\psi_{h}+\psi_{v}\right)\left(1-\cos\left(\frac{2\pi j}{n}\right)\cos\left(\frac{2\pi k}{m}\right)\right)\\ +w_{1,-1}H^{\prime }\left(\psi_{h}-\psi_{v}\right)\left(1-\cos\left(\frac{2\pi j}{n}\right)\cos\left(\frac{2\pi k}{m}\right)\right)\\ +w_{-1,-1}H^{\prime }\left(-\psi_{h}-\psi_{v}\right)\left(1-\cos\left(\frac{2\pi j}{n}\right)\cos\left(\frac{2\pi k}{m}\right)\right)\\ +w_{2,0}H^{\prime }\left(2\psi_{h}\right)\left(1-\cos\left(\frac{4\pi j}{n}\right)\right) \end{array}$$

(12) $$\begin{array}{l} +w_{-2,0}H^{\prime }(-2\psi_{h})\left(1-\cos\left(\frac{4\pi j}{n}\right)\right)\\ +w_{0,2}H^{\prime }(2\psi_{v})\left(1-\cos\left(\frac{4\pi k}{m}\right)\right)\\ +w_{0,-2}H^{\prime }(-2\psi_{v})\left(1-\cos\left(\frac{4\pi k}{m}\right)\right)]. \end{array}$$

Solutions will be stable whenever the real parts of the eigenvalues are negative.

In the following, we consider solutions when the coupling is symmetric in each direction (horizontal, vertical, and diagonal). In this case, *w*_±1, 0_ = *h*_1_, *w*_0, ±1_ = *v*_1_, *w*_±1, ±1_ = *d*, *w*_±2, 0_ = *h*_2_, and *w*_0, ±2_ = *v*_2_.

Then the real parts of the eigenvalues are


(13) $$\begin{array}{l} \;ℜ\left(\lambda_{jk}^{J}\right)\\ \;=-2\{h_{1}H_{odd^{\prime }}(\psi_{h})\left(1-\cos\left(\frac{2\pi j}{n}\right)\right)+v_{1}H_{odd^{\prime }}(\psi_{v})\left(1-\cos\left(\frac{2\pi k}{m}\right)\right)\\ \;+d\left[H_{odd^{\prime }}(\psi_{h}+\psi_{v})\left(1-\cos\left(\frac{2\pi k}{m}+\frac{2\pi j}{n}\right)\right)\right]\\ \;+d\left[H_{odd^{\prime }}(\psi_{h}-\psi_{v})\left(1-\cos\left(\frac{2\pi k}{m}-\frac{2\pi j}{n}\right)\right)\right]\\ \;+h_{2}H_{odd^{\prime }}(2\psi_{h})\left(1-\cos\left(\frac{4\pi j}{n}\right)\right)+v_{2}H_{odd^{\prime }}(2\psi_{v})\left(1-\cos\left(\frac{4\pi k}{m}\right)\right)\}, \end{array}$$

for 0 ≤ *j* ≤ *n*−1, 0 ≤ *k* ≤ *m*−1.

In summary, equations (12) and (13) give the eigenvalues for any cluster solution as described in Proposition 1 and hence can be used to determine their stability. Recall that such solutions are referred to as (*p*_*v*_, *p*_*h*_) *p*-cluster solutions, and have constant phase differences ψ_*h*_ and ψ_*v*_ between adjacent cells in the horizontal and vertical directions, respectively.

We now describe some special cases, which give rise to particular patterns of activity.

**Case 1 (Synchronous solution)**. For any values of *n* and *m*, the system admits the solution with ψ_*h*_ = ψ_*v*_ = 0. This corresponds to a 1-cluster solution (*p*_*h*_ = *p*_*v*_ = 1); hence all neurons are firing in phase. The eigenvalues in this case have


(14) $$\begin{array}{l} ℜ\left(\lambda_{jk}^{J}\right)=-2H_{odd^{\prime }}(0)[h_{1}\left(1-\cos\left(\frac{2\pi j}{n}\right)\right)+v_{1}\left(1-\cos\left(\frac{2\pi k}{m}\right)\right)\\ \;+2d\left(1-\cos\left(\frac{2\pi k}{m}\right)\cos\left(\frac{2\pi j}{n}\right)\right)\\ \;+h_{2}\left(1-\cos\left(\frac{4\pi j}{n}\right)\right)+v_{2}\left(1-\cos\left(\frac{4\pi k}{m}\right)\right)]. \end{array}$$

This solution will be stable if *H*′(0)>0.

**Case 2 (Diagonal Stripes)**. If *n* and *m* have a common factor *p*, then it is possible to have a solution where $\psi_{h}=\psi_{v}=\psi =\frac{2\ell \pi }{p}$, where *l* < *p* and gcd(*l, p*) = 1. This corresponds to a *p*-cluster solution (with *p*_*h*_ = *p*_*v*_ = *p*) where nearest neighbors (vertically and horizontally) are out of phase by ψ. In the 2D network this appears as a diagonal stripe pattern, where neurons are in-phase with their diagonal neighbors above-right and below-left. The eigenvalues in this case have


(15) $$\begin{array}{l} \;ℜ\left(\lambda_{jk}^{J}\right)\\ \;=-2\{H_{odd^{\prime }}(\psi )\left[h_{1}\left(1-\cos\left(\frac{2\pi j}{n}\right)\right)+v_{1}\left(1-\cos\left(\frac{2\pi k}{m}\right)\right)\right]\\ +d\left[{H^{\prime }}_{odd}(2\psi )\left(1-\cos\left(\frac{2\pi k}{m}+\frac{2\pi j}{n}\right)\right)+H_{odd^{\prime }}(0)\left(1-\cos\left(\frac{2\pi k}{m}-\frac{2\pi j}{n}\right)\right)\right]\\ \;{H^{\prime }}_{odd}(2\psi )\left[h_{2}\left(1-\cos\left(\frac{4\pi j}{n}\right)\right)+v_{2}\left(1-\cos\left(\frac{4\pi k}{m}\right)\right)\right]\}. \end{array}$$

There is also a *p*-cluster solution with $\psi_{h}=\psi =\frac{2\ell \pi }{p}$ and ψ_*v*_ = 2π−ψ. In the 2D network this appears as a diagonal stripe pattern, where neurons are in-phase with their diagonal neighbors above-left and below-right. The eigenvalues in this case are


(16) $$\begin{array}{l} ℜ\left(\lambda_{jk}^{J}\right)=-2\{H_{odd^{\prime }}(\psi )\left[h_{1}\left(1-\cos\left(\frac{2\pi j}{n}\right)\right)+v_{1}\left(1-\cos\left(\frac{2\pi k}{m}\right)\right)\right]\\ +d\left[H_{odd^{\prime }}(0)\left(1-\cos\left(\frac{2\pi k}{m}+\frac{2\pi j}{n}\right)\right)+{H^{\prime }}_{odd}(2\psi )\left(1-\cos\left(\frac{2\pi k}{m}-\frac{2\pi j}{n}\right)\right)\right]\\ \;{H^{\prime }}_{odd}(2\psi )\left[h_{2}\left(1-\cos\left(\frac{4\pi j}{n}\right)\right)+v_{2}\left(1-\cos\left(\frac{4\pi k}{m}\right)\right)\right]\}. \end{array}$$

**Case 3 (Horizontal Stripes)**. If *p* is a factor of *m*, then the system admits a solution with ψ_*h*_ = 0 and $\psi_{v}=\frac{2\ell \pi }{p}$, where ℓ < *p* and gcd(ℓ, *p*) = 1. This is a *p*-cluster solution (with *p*_*h*_ = 1, *p*_*v*_ = *p*) where neurons are synchronized with all their horizontal neighbors and have phase difference ψ_*v*_ with their nearest vertical neighbors. Thus, these appear as horizontal stripes. The eigenvalues in this case have


(17) $$\begin{array}{l} \;ℜ\left(\lambda_{jk}^{J}\right)\\ =-2\{H_{odd^{\prime }}(\psi_{v})\left[v_{1}\left(1-\cos\left(\frac{2\pi k}{m}\right)\right)+2d\left(1-\cos\left(\frac{2\pi k}{m}\right)\cos\left(\frac{2\pi j}{n}\right)\right)\right]\\ \;+H_{odd^{\prime }}(2\psi_{v})v_{2}\left(1-\cos\left(\frac{4\pi k}{m}\right)\right)\\ \;+H_{odd^{\prime }}(0)\left[h_{1}\left(1-\cos\left(\frac{2\pi j}{n}\right)\right)+h_{2}\left(1-\cos\left(\frac{4\pi j}{n}\right)\right)\right]\}. \end{array}$$

**Case 4 (Vertical Stripes)**. Similarly, if *p* is a factor of *n*, then the system admits a solution with ψ_*v*_ = 0 and $\psi_{h}=\frac{2\ell \pi }{p}$, where ℓ < *p* and gcd(ℓ, *p*) = 1. The eigenvalues will be the same as in Case 3 with the roles of *v*_1_ and *h*_1_ swapped and those of *v*_2_ and *h*_2_ swapped. This is a *p*-cluster solution (with *p*_*h*_ = *p, p*_*v*_ = 1) where neurons are synchronized with all their vertical neighbors and have phase difference ψ_*h*_ with their nearest horizontal neighbors. Thus, these appear as vertical stripes.

**Figure 2:**
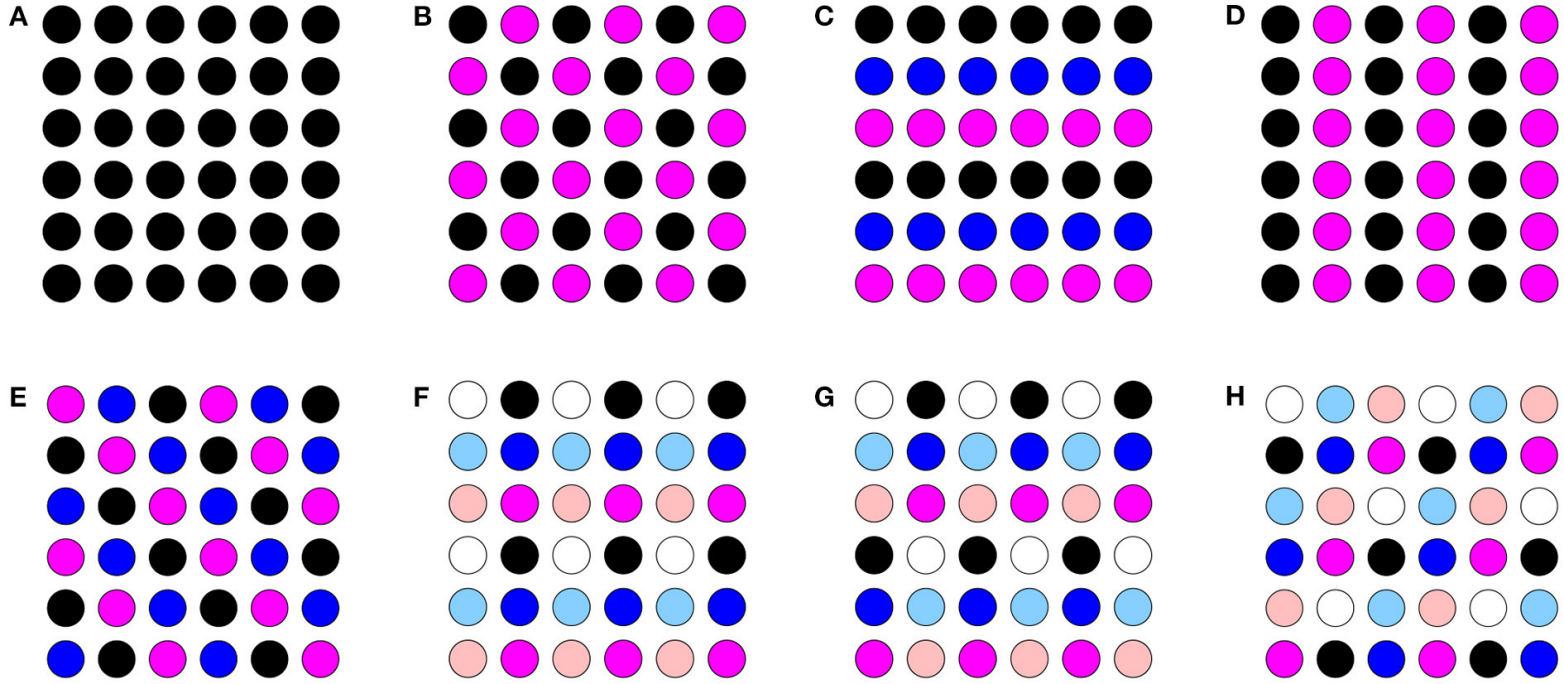
Illustration of cluster solutions in a 6 × 6 network. Circles that are the same color represent neurons that are in the same cluster. **(A)** synchronous solution. **(B)** 2-cluster diagonal stripe solution. **(C)** 3-cluster horizontal stripe solution. **(D)** 2-cluster vertical stripe solution. **(E)** 3-cluster diagonal stripe solution. **(F)** (2, 3) 6-cluster solution. **(G)** (2, 6) 6-cluster solution. **(H)** (3, 6) 6-cluster solution.

Figure 2 illustrates some of the possible cluster solutions that exist in a 6 × 6 network. In this figure, circles of the same color correspond to neurons that spike synchronously (in-phase), and thus belong to the same cluster. Figure 2A shows the synchronous solution. Figure 2B shows a 2-cluster diagonal stripe solution of the first type, i.e., with ψ_*h*_ = ψ_*v*_ = π. Figure 2C shows a 3-cluster horizontal stripe solution. Figure 2D shows a 2-cluster vertical stripe solution. Figure 2E shows a 3-cluster diagonal stripe solution of the second type, i.e., with $\psi_{h}=\frac{2\pi }{3}$, $\psi_{v}=\frac{4\pi }{3}$. Figure 2F shows a (2, 3) 6-cluster solution, corresponding to $\psi_{h}=\pi ,\;\psi_{v}=\frac{2\pi }{3}$. Each row splits into two clusters and each column into three, giving six clusters overall in the network. Similarly Figure 2G shows a (2, 6) 6-cluster solution, corresponding to $\psi_{h}=\pi ,\;\psi_{v}=\frac{\pi }{3}$ and Figure 2H shows a (3, 6) 6-cluster solution, corresponding to $\psi_{h}=\frac{2\pi }{3},\;\psi_{v}=\frac{\pi }{3}$.

## 4. Applications and numerical results

In this section, we consider existence and stability of cluster solutions in the 2D, biophysical inhibitory network of Wang-Buzsaki neurons described in Section 2.2.

### 4.1. Homogeneous first nearest neighbor coupling—effect of diagonal coupling

**Figure 3:**
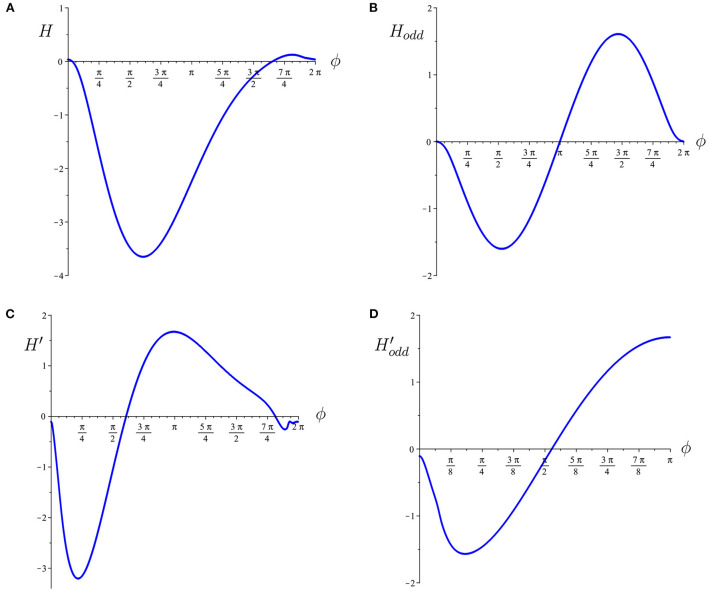
**(A)** The interaction function for the phase model, *H*(ϕ). **(B)** The odd part of the interaction function, *H*_*odd*_(ϕ). Both are numerically computed for the biophysical inhibitory network of Wang-Buzsaki neurons (calculated using XPPAUT). **(C,D)** The derivatives of these functions, calculated using finite differences. For computation of stability conditions, we note that $H_{odd}^{\prime }\left(0\right)\approx -0.11,\;H_{odd}^{\prime }\left(\frac{\pi }{3}\right)\approx -1.14,\;H_{odd}^{\prime }\left(\frac{\pi }{2}\right)\approx -0.18,\;H_{odd}^{\prime }\left(\frac{2\pi }{3}\right)\approx 0.78,\;H_{odd}^{\prime }\left(\pi \right)\approx 1.67$.

We first consider the case of first nearest neighbor coupling only, so *h*_2_ = *v*_2_ = 0. To study the effect of diagonal coupling with otherwise homogeneous coupling strengths, we choose either *h*_1_ = *v*_1_>0, *d* = 0 or *h*_1_ = *v*_1_ = *d*>0. Note that these cases correspond to each neuron being coupled to four (left, right, up, down) or eight (left, right, up, down, four diagonal) neighboring neurons, respectively. In our model, $H_{odd}^{\prime }\left(\phi \right)<0$ for $\phi \in \left(0,\frac{17\pi }{32}\right)\cup \left(\frac{47\pi }{32},2\pi \right)$ and $H_{odd}^{\prime }\left(\phi \right)>0$ for $\phi \in \left[\frac{17\pi }{32},\frac{47\pi }{32}\right]$ (Figure 3). In the following, we consider the stability of the solutions described in Section 3 above.

#### 4.1.1. Diagonal stripes

As shown in Section 3, if *n* and *m* are divisible by *p*, the system admits *p*-cluster diagonal stripe solutions with phase differences ψ_*h*_ = ψ_*v*_ = ψ and ψ_*h*_ = ψ, ψ_*v*_ = 2π−ψ. Consideration of Equations (15), (16) with *h*_2_ = *v*_2_ = 0 and *d* = 0 shows that these diagonal stripe solutions will be stable if $H_{odd}^{\prime }\left(\psi \right)>0$ and unstable if $H_{odd}^{\prime }\left(\psi \right)<0$. For example, the 2- and 3-cluster diagonal stripe solutions (corresponding to ψ = π and $\psi =\frac{2\pi }{3}$ or $\frac{4\pi }{3}$, respectively) will be stable if they exist in a given network, while the 4- and 6-cluster solutions ($\psi =\frac{\pi }{2}$ or $\frac{3\pi }{2}$ and $\psi =\frac{\pi }{3}$ or $\frac{5\pi }{3}$, respectively) will be unstable.

Setting *d* > 0 in Equations (15), (16) shows that the addition of diagonal coupling can destabilize a stable diagonal stripe solution if $\min\left(h_{1},v_{1}\right)H_{odd}^{\prime }\left(\psi \right)+d\left(H_{odd}^{\prime }\left(2\psi \right)+H_{odd}^{\prime }\left(0\right)\right)<0$. For example, the 2-cluster diagonal stripe solution can be destabilized in our network model if


(18) $$\begin{array}{l} d>-\frac{H_{odd}\left(\pi \right)}{2H_{odd}^{\prime }\left(0\right)}\;\min\left(h_{1},v_{1}\right)\approx 7.59\;\min\left(h_{1},v_{1}\right), \end{array}$$

but the 3-cluster diagonal stripe solutions cannot be destabilized as $H_{odd}^{\prime }\left(\frac{4\pi }{3}\right)+H_{odd}^{\prime }\left(0\right)>0.$ Diagonal cluster solutions that are unstable with *d* = 0 need to be considered individually. Analysis of the eigenvalues (15)-(16) shows that with homogeneous coupling (*h*_1_ = *v*_1_ = *d*), the 4-cluster and 6-cluster diagonal stripe solutions are unstable in the 4 × 4 and 6 × 6 networks, respectively.

#### 4.1.2. Horizontal/vertical stripes

As shown in Section 3, if *m* is divisible by *p*, the system admits a *p*-cluster horizontal stripe solution with phase differences ψ_*h*_ = 0 and ψ_*v*_≠0. Since $H_{odd}^{\prime }\left(0\right)<0$ for our model, if *v*_2_ = *h*_2_ = 0 and *d* = 0 in Equation (17) the eigenvalues $\lambda_{j0}^{J}$ will have positive real part. So without diagonal coupling, we expect the horizontal stripe solutions (and similarly the vertical stripe solutions) to be unstable. However, if *d*>0, then these eigenvalues have real part


$$ℜ\left(\lambda_{j0}^{J}\right)=-2\left[2dH_{odd}^{\prime }\left(\psi_{v}\right)+h_{1}H_{odd}^{\prime }\left(0\right)\right]\left(1-\cos\left(\frac{2\pi j}{n}\right)\right).$$

Thus, if


(19) $$\begin{array}{l} 2dH_{odd}^{\prime }\left(\psi_{v}\right)+h_{1}H_{odd}^{\prime }\left(0\right)>0, \end{array}$$

the *p*-cluster horizontal stripe solutions may be stable. From Figure 3, it is clear that this will only be possible for a restricted range $\sim \left(\frac{\pi }{2},\frac{3\pi }{2}\right)$ of values of ψ_*v*_. In particular, diagonal coupling may be able to stabilize the 2-cluster and 3-cluster horizontal stripe solutions, but not the 4-cluster or 6-cluster ones. Detailed analysis of the eigenvalues shows that for homogeneous coupling, *h*_1_ = *v*_1_ = *d*, the 2-cluster solution is stable in the 4 × 4 network and both the 2- and 3-cluster solutions are stable in the 6 × 6 network. A similar analysis applies to the vertical stripe solutions.

#### 4.1.3. Other cluster solutions

As described in Proposition 3, if *p*_*h*_ divides *n* and *p*_*v*_ divides *m*, then the system admits a (*p*_*h*_, *p*_*v*_) *p*-cluster solution with *p* = lcm(*p*_*h*_, *p*_*v*_). Here, we focus on the cases with *p*_*h*_≠*p*_*v*_ and min{*p*_*h*_, *p*_*v*_}>1. If a network is symmetric (*n* = *m*) we may assume that *p*_*h*_<*p*_*v*_, as similar stability results for *p*-cluster solutions with *p*_*h*_ > *p*_*v*_ can be obtained by interchanging the horizontal and vertical directions.

In the case of nearest neighbor coupling only (i.e., *v*_2_ = *h*_2_ = *d* = 0), we know from Equation (14) that this *p*-cluster is stable if $H_{odd}^{\prime }\left(\psi_{h}\right)>0$ and $H_{odd}^{\prime }\left(\psi_{v}\right)>0$, and this solution is unstable if $H_{odd}^{\prime }\left(\psi_{h}\right)<0$ or $H_{odd}^{\prime }\left(\psi_{v}\right)<0$. For instance, suppose that *p*_*h*_ = 2 (for which *n* has to be even), then ψ_*h*_ = π and $H_{odd}^{\prime }\left(\pi \right)>0$. In 2 × 4 or 4 × 4 networks, *p*_*v*_ = 1, 2 or 4. Since we assume that *p*_*h*_ ≠ *p*_*v*_ and *p*_*v*_ > 1, *p*_*v*_ = 4. In this case, we have a (2, 4) 4-cluster solution (since lcm(2, 4) = 4). However, this solution will be unstable as $H_{odd}^{\prime }\left(\psi_{v}\right)<0$ for all possible ψ_*v*_. Similarly, in 2 × 6, 4 × 6 or 6 × 6 networks, we must have *p*_*v*_ = 3 or 6. If *p*_*v*_ = 3, it leads to a (2, 3) 6-cluster solution, and this solution is stable, since $H_{odd}^{\prime }\left(\psi_{v}\right)>0$ for all the possible ψ_*v*_. If *p*_*v*_ = 6, it leads to a (2, 6) 6-cluster solution. However, this solution will be unstable as $H_{odd}^{\prime }\left(\psi_{v}\right)<0$ for all possible ψ_*v*_. In 6 × 6 networks we can also have a (3, 6) 6-cluster solution which is similarly unstable.

With the diagonal coupling, *d* > 0, a stable (or unstable) solution can be maintained if $H_{odd}^{\prime }\left(\psi_{h}\pm \psi_{v}\right)>0$ (or $H_{odd}^{\prime }\left(\psi_{h}\pm \psi_{v}\right)<0$). Otherwise adding diagonal coupling may change the stability. In 4 × 4 networks, regarding the (2, 4) 4-cluster solution discussed before, adding diagonal neighbors could not reverse the instability as $H_{odd}^{\prime }\left(\psi_{h}\pm \psi_{v}\right)<0$. In contrast, in 6 × 6 networks, for the (2, 3) 6-cluster solution that is stable without diagonal coupling, adding diagonal neighbors tends to destabilize the solution, and the stability could be lost if *d* ≫ max{*h*_1_, *v*_1_}. For the (2, 6) 6-cluster solutions, which are unstable without diagonal coupling, including diagonal neighbors tends to stabilize the solution, and may change the solution from unstable to stable if *d* ≫ *v*_1_. For the (3, 6) 6-cluster solutions adding diagonal neighbors brings in both stabilizing and destabilizing terms. Further analysis of the eigenvalues with homogeneous coupling (*h*_1_ = *v*_1_ = *d*) shows that in the 6 × 6 network the (2, 3) solution is stabilized, the (2, 6) is destabilized and the (3, 6) solution remains unstable.

We illustrate the stability of these multiple cluster solutions in numerical solutions of inhibitory networks of Wang-Buzsaki neurons. In these simulations, synaptic strength is homogeneous at the maximum conductance level *h*_1_ = *v*_1_ = *g*_*syn*_, and *d* = *g*_*syn*_ when it is non-zero.

#### 4.1.4. Numerical simulations in 4 × 4 networks

**Figure 4:**
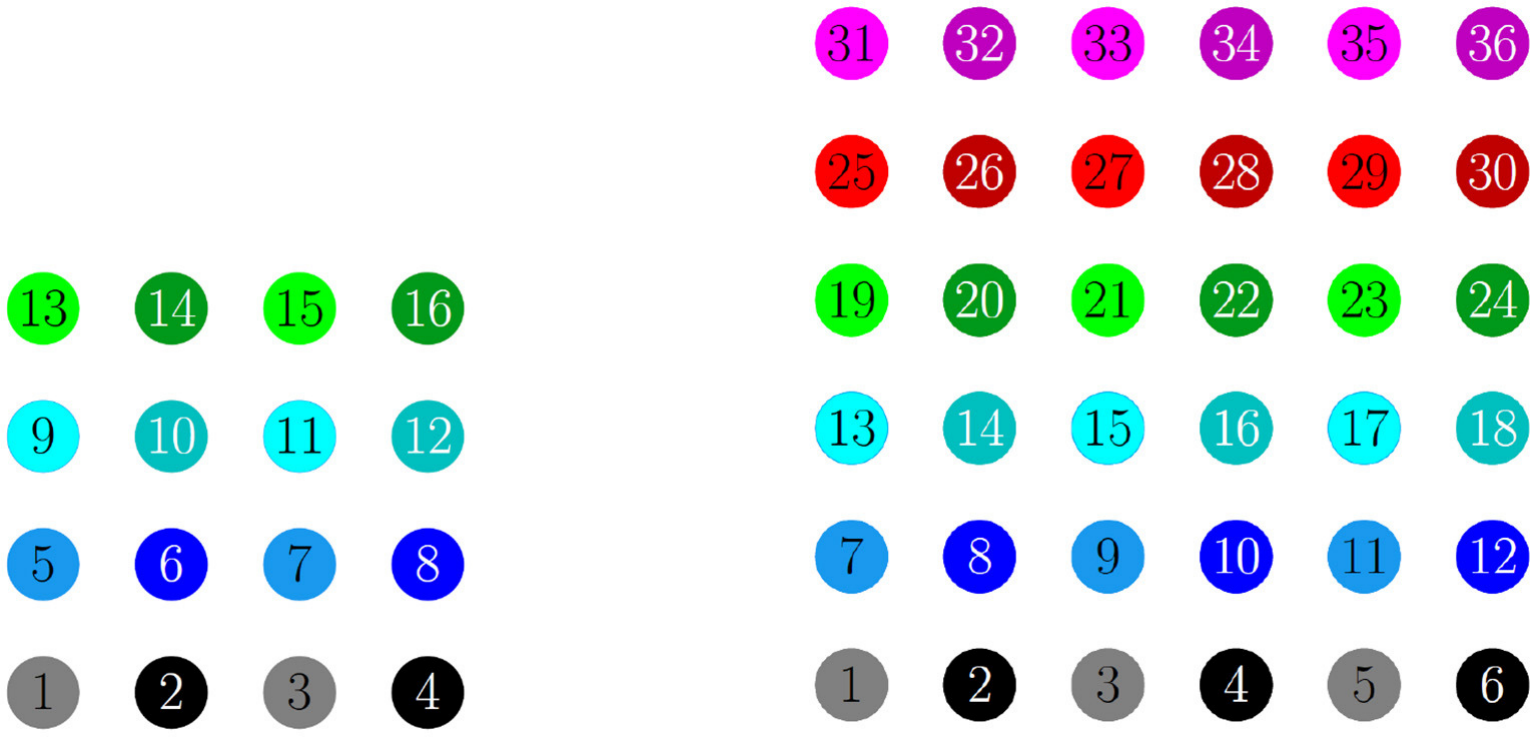
Illustration of the 2D configuration of cells for numerical simulations: 4 × 4 network **(left)** and 6 × 6 network **(right)**. In both, the cells are numbered consecutively across rows of the network lattice, and odd numbered cells are a lighter shade compared to even numbered cells. In the figures that follow, the raster plots use the same colors.

**Figure 5:**
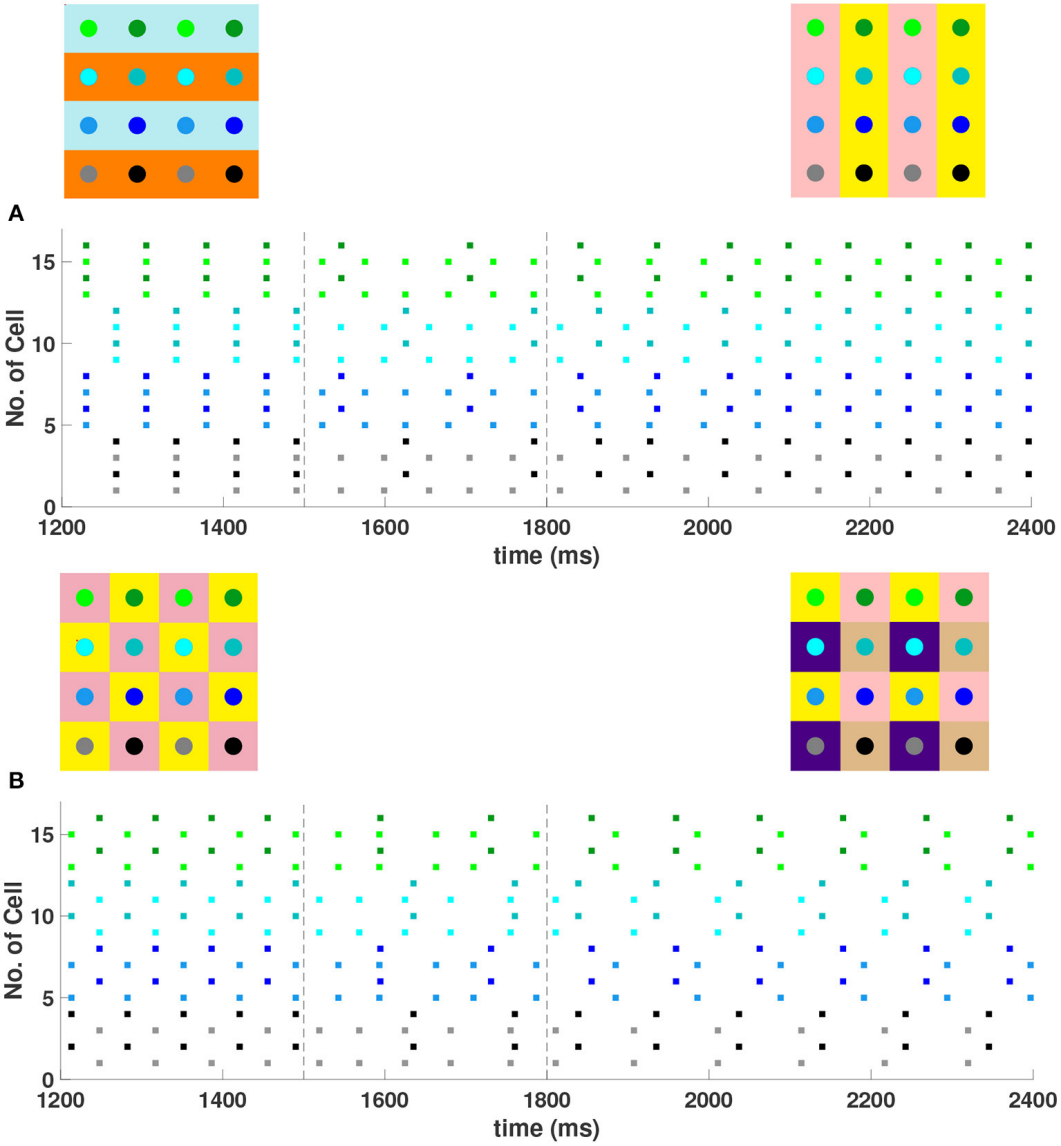
Multiple stable cluster solutions in 4 × 4 inhibitory neural networks of Wang-Buzsaki model neurons. In the network cells are coupled to their first nearest neighbors in the horizontal, vertical, and diagonal directions (*h*_1_ = *v*_1_ = *d* = *g*_*syn*_, *h*_2_ = *v*_2_ = 0) **(A)** Network was initiated in the 2-cluster horizontal stripe solution and a transient perturbation (additional applied current to a subset of cells) was given over the time interval *t*∈[1500, 1800] (between dashed vertical lines) which switches the solution to the 2-cluster vertical stripe solution. **(B)** Network was initiated in the 2-cluster diagonal stripe solution and a similar transient perturbation to a different subset of cells switched the solution to the stable 4-cluster solution. Above each raster plot, we show the clusters in 2D at the beginning and end. Colors inside the circle correspond to the colors in the raster plot. Circles with the same background color represent neurons that are in the same cluster.

For a 4 × 4 network with *h*_1_ = *v*_1_ = *d* = *g*_*syn*_, 4 different cluster solutions are stable: 2-cluster horizontal, vertical, and diagonal stripe solutions, and a 4-cluster solution in which groups of 4 cells fire synchronously with a $\frac{\pi }{2}$ phase difference between successively firing groups. We show the numbering and coloring of our 4 × 4 and 6 × 6 networks in Figure 4. When the cells are numbered consecutively across rows of the 4 × 4 network lattice, the clusters in this 4-cluster solution consist of cells {1, 3, 9, 11}, {2, 4, 10, 12}, {6, 8, 14, 16}, and {5, 7, 13, 15}. This solution differs from those discussed so far since the horizontal and vertical phase differences ψ_*h*_ and ψ_*v*_ are not the same for all cells but instead alternate between $\frac{\pi }{2}$ and $-\frac{\pi }{2}$ between neighboring cells. The stability of these 4 solutions in the same network is demonstrated in Figure 5. In each simulation, the network is initialized in one of the stable solutions (A: 2-cluster horizontal stripe; B: 2-cluster diagonal stripe) and a transient perturbation, in the form of increased external current applied to a subset of cells, switches the network to a different stable solution (A: 2-cluster vertical stripe; B: 4-cluster solution). The corresponding 2D clusters are shown above each raster plot. If the diagonal coupling is removed (*d* = 0) other simulations (not shown) indicate that the 2-cluster horizontal stripe solution becomes unstable, while the 2-cluster diagonal stripe solution remains stable.

#### 4.1.5. Numerical simulations in 6 × 6 networks

**Figure 6:**
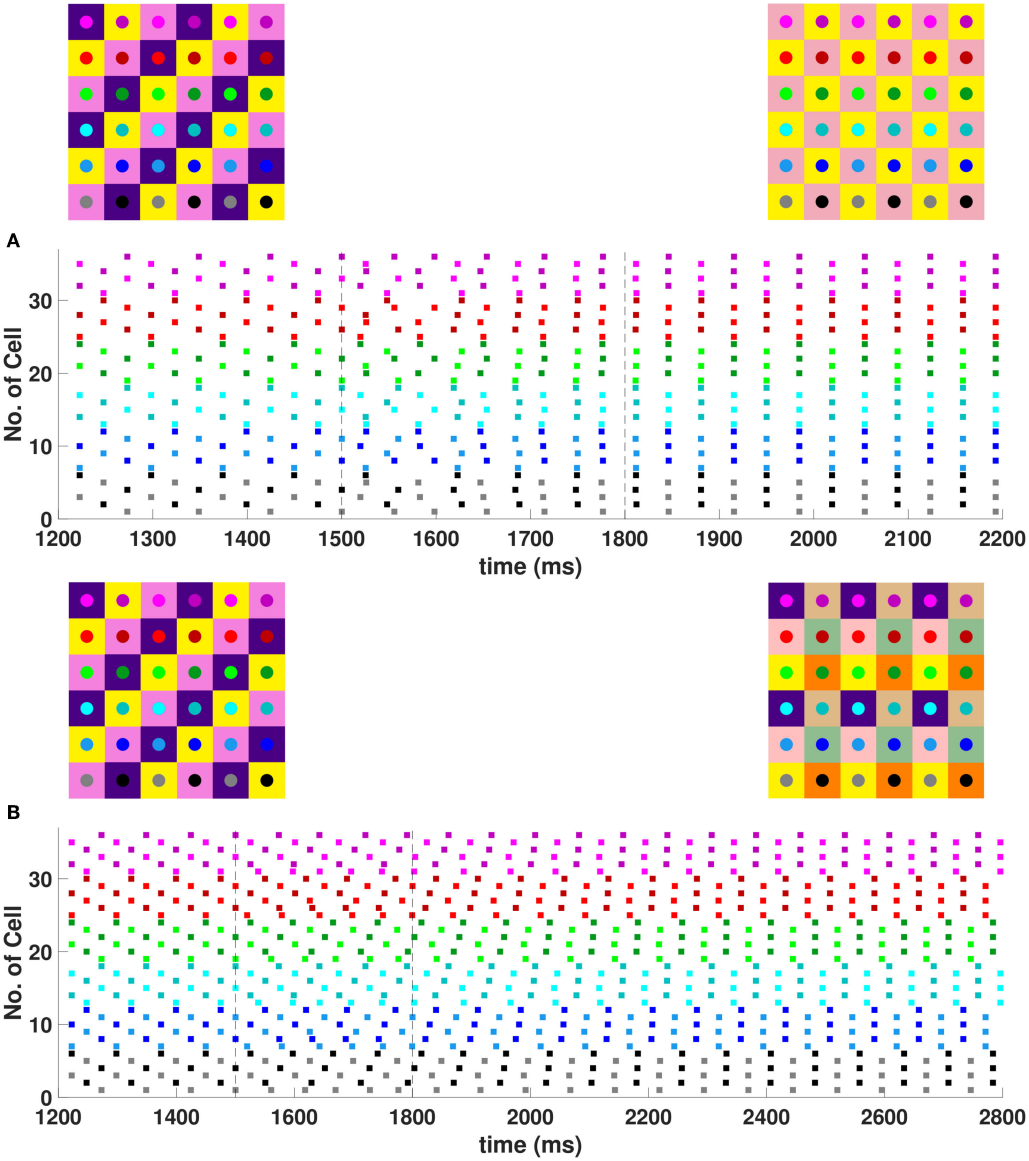
Multiple stable cluster solutions in 6 × 6 inhibitory neural network. Cells are coupled to their first nearest neighbors in the horizontal and vertical directions only (*h*_1_ = *v*_1_ = *g*_*syn*_, *d* = *h*_2_ = *v*_2_ = 0) **(A)** Network is initialized in a stable 3-cluster diagonal stripe solution. A transient perturbation to a subset of cells, given at *t*∈[1500, 1800] ms, switches the solution to a stable 2-cluster diagonal stripe solution. **(B)** Network is initialized in a stable 3-cluster diagonal stripe solution and a transient perturbation to a different subset of cells switches the solution to a stable (2, 3) 6-cluster solution. Above each raster plot, we show the clusters in 2D at the beginning and end. Colors inside the circle correspond to the colors in the raster plot. Circles with the same background color represent neurons that are in the same cluster.

In the 6 × 6 network, 2-, 3-, and 6-cluster solutions are stable. For example, without diagonal coupling in the network (*d* = 0), both 2- and 3-cluster diagonal stripe solutions are stable and transient perturbations can switch the network between these stable solutions (Figure 6A). In this simulation, the network is initialized in the 3-cluster diagonal stripe solution and at time *t*∈[1500, 1800] ms the applied current is transiently increased to a subset of cells. This perturbation switches the solution to the stable 2-cluster diagonal stripe solution. A perturbation of the same magnitude to a different subset of cells switches the 3-cluster diagonal stripe solution to a (2, 3) 6-cluster solution such that ψ_*h*_ = π and $\psi_{v}=\frac{2\pi }{3}$ (Figure 6B). The corresponding 2D clusters are shown above each raster plot. In the (2, 3) 6-cluster solution, cells across rows break into 2 clusters while cells along columns separate into 3 clusters, as illustrated in Figure 2F. In this simulation, the evolution to the stable (2, 3) 6-cluster solution took longer following the transient perturbation, compared to the evolution to the 2-cluster diagonal stripe solution. Generally, we expect that the evolution time to a new stable solution will depend on the specific perturbation given to induce the switch of solutions.

**Figure 7:**
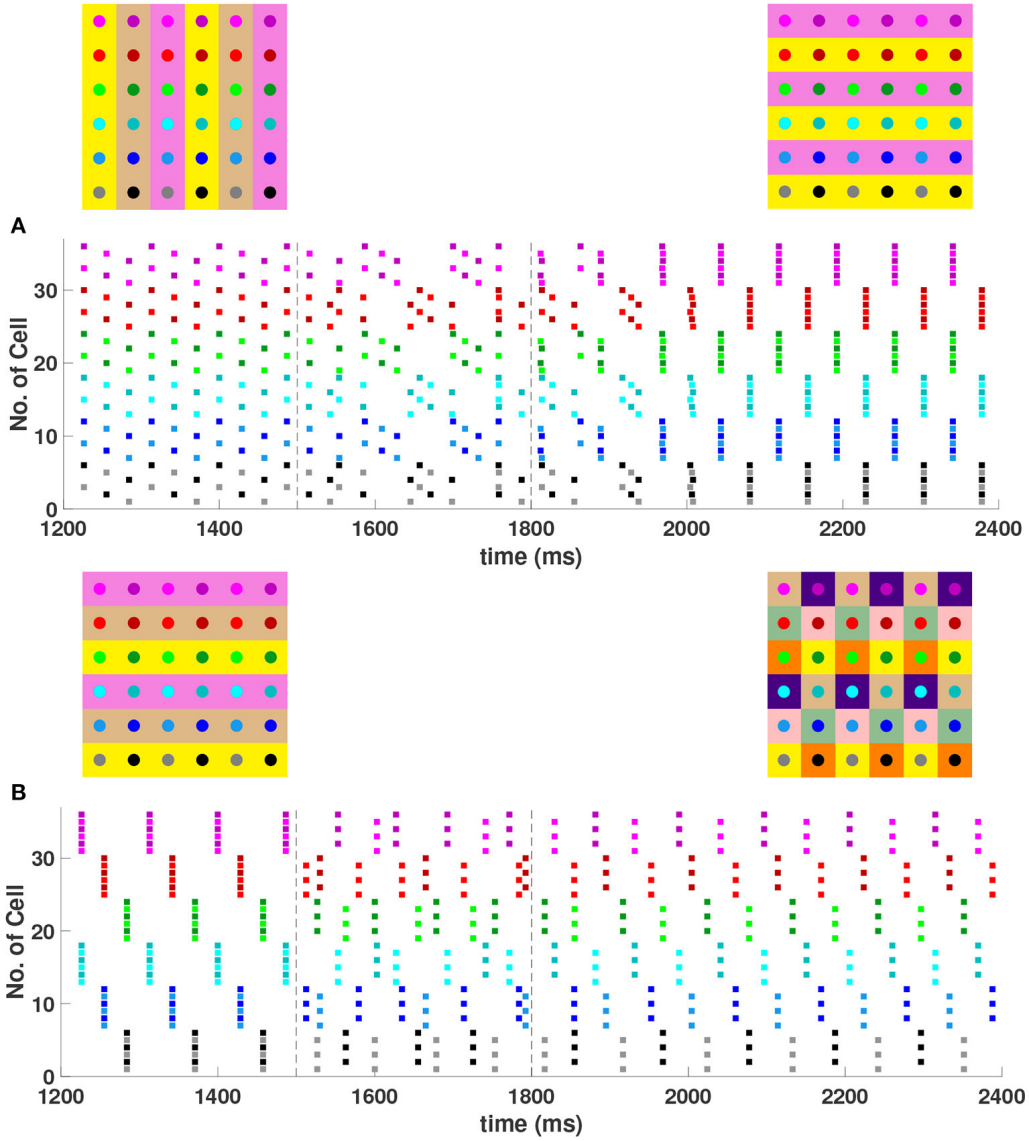
Multiple stable cluster solutions in the 6 × 6 network. Cells are coupled to their first nearest neighbors in the horizontal, vertical, and diagonal directions (*h*_1_ = *v*_1_ = *d* = *g*_*syn*_, *h*_2_ = *v*_2_ = 0). **(A)** Network is initialized in a stable 3-cluster vertical stripe solution. A transient perturbation to a subset of cells, given at *t*∈[1500, 1800] ms, switches the solution to a stable 2-cluster horizontal stripe solution. **(B)** Network is initialized in a stable 3-cluster horizontal stripe solution and a transient perturbation to a different subset of cells switches the solution to a stable (2, 6) 6-cluster solution.

In the 6 × 6 network with the addition of diagonal first nearest neighbor coupling (i.e., *d* = *g*_*syn*_), both 2- and 3-cluster horizontal and vertical stripe solutions are stable, and transient perturbations can switch the network between these solutions (Figure 7). For example, for a network in the 3-cluster vertical stripe solution, a transient perturbation to a subset of cells switches the solution to the stable 2-cluster horizontal stripe solution (Figure 7A). In the same network initialized in the 3-cluster horizontal stripe solution, a transient perturbation to a different subset of cells switches the network to a stable (2, 6) 6-cluster solution with ψ_*h*_ = π and $\psi_{v}=\frac{\pi }{3}$ (Figure 7B). In this solution, cells across rows break into 2 clusters while cells along columns separate into 6 clusters, as illustrated in Figure 2G.

### 4.2. Effect of second nearest neighbor homogeneous coupling

Now we study the effect on the stability of the cluster solutions of adding second nearest coupling with homogeneous coupling strengths, i.e., *h*_2_ = *v*_2_ = *d* = *h*_1_ = *v*_1_>0. For notational simplicity, we denote the first nearest neighbor coupling considered in the last section as 8 nearest neighbor coupling.

#### 4.2.1. Diagonal stripes

As discussed in Section 4.1, if both *m* and *n* are even, the system admits a 2-cluster diagonal stripe solution with constant phase difference ψ_*h*_ = ψ_*v*_ = π. Adding the second nearest neighbor coupling tends to destabilize the diagonal stripes since $H_{odd}^{\prime }\left(2\psi_{h}\right)=H_{odd}^{\prime }\left(2\psi_{v}\right)=H_{odd}^{\prime }\left(0\right)<0$.

If *m* and *n* are divisible by 3, there is a 3-cluster diagonal stripe solution. In our model, this solution is stable with 8 nearest neighbors coupling. Adding second nearest neighbor coupling will increase the stability as $H_{odd}^{\prime }\left(2\psi_{h}\right)=H_{odd}^{\prime }\left(2\psi_{v}\right)=H_{odd}^{\prime }\left(2\pi /3\right)>0$. Thus, the 3-cluster diagonal solution will be stable with any strengths of second nearest neighbor coupling.

If *n* and *m* are divisible by 6, there is a 6-cluster diagonal stripe solution. In our model, this solution is unstable with 8 nearest neighbors coupling. Adding second nearest neighbor coupling may increase ($\psi_{h}=\frac{\pi }{3}$) or decrease ($\psi_{h}=\frac{5\pi }{3}$) the stability.

Further analysis of the eigenvalues in Equations (15), (16) for the 6 × 6 network with homogeneous coupling (*h*_2_ = *v*_2_ = *d* = *h*_1_ = *v*_1_>0), shows that the 2-cluster diagonal stripe solutions remain stable, the 3-cluster solutions remain stable and the 6-cluster solutions remain unstable.

#### 4.2.2. Horizontal/vertical stripes

If *m* is divisible by *p*, the system admits a *p*-cluster horizontal stripe solution with phase difference ψ_*h*_ = 0 and ψ_*v*_≠0. Including the second nearest neighbor coupling may or may not change the stability of horizontal stripe solutions. For example, in 6 × 6 networks, there is a 6-cluster horizontal stripe solution with $\psi_{v}=\frac{\pi }{3}$ or $\frac{5\pi }{3}$. This solution is unstable regardless of the presence of the second nearest neighbor coupling since there is always an eigenvalue with positive real part (From Equation 17, $ℜ\left(\lambda_{0k}^{J}\right)>0,\;k=1,2,\ldots ,5$). The same network also admits 2-cluster and 3-cluster horizontal stripe solutions, which are stable with 8 nearest neighbor coupling (see Section 4.1). Evaluation of the eigenvalues in Equation (17) shows that homogeneous second nearest neighbor coupling (*h*_2_ = *v*_2_ = *h*_1_ = *v*_1_ = *d*) cannot destabilize either of these solutions. However, we shall see in the next section that heterogeneous coupling could destabilize these solutions, e.g., if *h*_2_ ≫ *v*_2_. Similar results are expected for vertical stripe solutions by swapping the horizontal and vertical directions.

#### 4.2.3. Other cluster solutions

If a (*p*_*h*_, *p*_*v*_) *p*-cluster solution is stable with 8 nearest neighbors coupling, then adding the second nearest neighbors can strengthen the stability if $H_{odd}^{\prime }\left(2\psi_{h}\right)>0$ and $H_{odd}^{\prime }\left(2\psi_{v}\right)>0$ but tends to destabilize the solution if $H_{odd}^{\prime }\left(2\psi_{h}\right)<0$ or $H_{odd}^{\prime }\left(2\psi_{v}\right)<0$. On the other hand, if a *p*-cluster solution with 8 nearest neighbors coupling is unstable, including the second nearest neighbors may change the stability. For instance, consider the (2, 3) 6-cluster solution in 6 × 6 networks. This solution could be stable in the absence of second nearest neighbor coupling if *d*≪min{*h*_1_, *v*_1_} but could be destabilized by adding second nearest neighbors if *v*_2_ ≫ max{*v*_1_, *h*_1_}. Evaluation of the eigenvalues in Equation (13) shows that homogeneous second nearest neighbor coupling (*h*_2_ = *v*_2_ = *h*_1_ = *v*_1_ = *d*) does not change the stability of any of the (*p*_*h*_, *p*_*v*_) 6-cluster solutions in the 6 × 6 network.

#### 4.2.4. Numerical simulations in 6 × 6 networks

**Figure 8:**
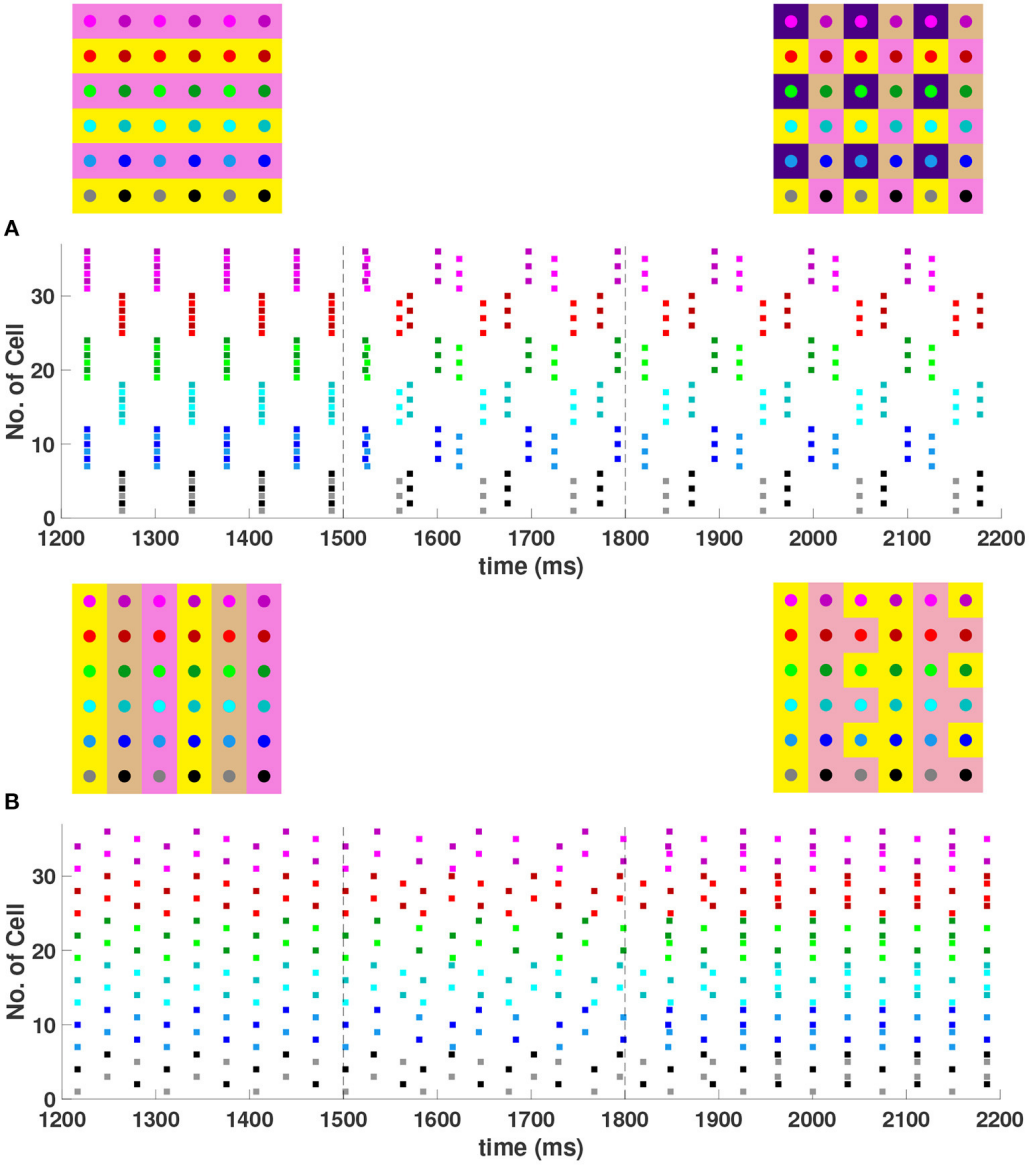
Stable cluster solutions in the 6 × 6 network. Cells are coupled to their first nearest neighbors in the horizontal, vertical, and diagonal directions, and to their second nearest neighbors in the horizontal and vertical directions (*h*_1_ = *v*_1_ = *d* = *h*_2_ = *v*_2_ = *g*_*syn*_). **(A)** Network is initialized in a stable 2-cluster horizontal stripe solution. A transient perturbation to a subset of cells, given at *t*∈[1500, 1800] ms, switches the solution to a stable 4-cluster solution. **(B)** Transition from a stable 3-cluster vertical stripe solution to a stable 2-cluster solution after the similar transient perturbation is applied.

When the coupling to the second nearest neighbors in horizontal and vertical directions are added to the 6 × 6 network (*h*_2_ = *v*_2_ = *g*_*syn*_), $\text{2}\bar{/}$, $\text{3}\bar{/}$, and 4-cluster solutions are stable and transient perturbations can switch the network between these stable solutions (Figure 8). In Figure 8A, the network is initialized in a stable 2-cluster horizontal stripe solution. A transient perturbation to a certain subset of cells switches the 2-cluster horizontal stripe solution to a stable 4-cluster solution in which groups of 9 cells fire synchronously with a $\frac{\pi }{2}$ phase difference between successively firing groups. Similar to the 4-cluster solution shown in Figure 5B, this solution differs from those considered in Section 3 since the horizontal and vertical phase differences ψ_*h*_ and ψ_*v*_ alternate between $\frac{\pi }{2}$ and $-\frac{\pi }{2}$ between neighboring cells. In Figure 8B, the same network is initialized in the 3-cluster vertical stripe solution and a transient perturbation to a subset of cells switches this solution to a stable 2-cluster solution with non-uniform phase differences between neighboring cells. Thus, some cells have constant ψ_*h*_ and ψ_*v*_, but other cells do not.

When the diagonal coupling is set to zero (*d* = 0) while keeping all other couplings non-zero in the 6 × 6 network, numerical simulation confirms that the 2-cluster horizontal stripe solution shown in Figure 8A becomes unstable, as predicted by evaluation of the eigenvalues in Equation (15) with *n* = *m* = 6, ψ_*h*_ = 0 and ψ_*v*_ = π.

### 4.3. Heterogeneous coupling strengths

The numerical simulations discussed so far have considered *h*_1_ = *v*_1_ = *d* = *h*_2_ = *v*_2_ for simplicity. However, our derived expressions for the eigenvalues e.g., Equation (13), do not depend on this assumption. We will consider the implications of this more general expression in this section.

First, we note that if the coupling strengths are slightly perturbed from this homogeneous case, then we don't expect that the stability should change. To see this, note that the eigenvalues in Equation 13 are linear in all the coupling strengths. So if the coupling strengths are perturbed from the homogeneous case, i.e., $h_{1}=A+B{ĥ}_{1},\;v_{1}=A+B{\hat{v}}_{1},\;d=A+B\hat{d},h_{2}=A+B{ĥ}_{2},\;v_{2}=A+B{\hat{v}}_{2}$, then the eigenvalues satisfy


$$ℜ\left(\lambda_{jk}^{J}\right){|}_{h_{1},v_{1},\ldots }=ℜ\left(\lambda_{jk}^{J}\right){|}_{A,A,\ldots }+B\;ℜ\left(\lambda_{jk}^{J}\right){|}_{{ĥ}_{1},\hat{v_{1}},\ldots }$$

So if *B* is small enough the change in the eigenvalue will be small enough that the real part should not change sign.

If the coupling strengths are changed significantly, of course, the stability of solutions may change. In the rest of this section we use the stability conditions in Section 3 to study how introducing strongly heterogeneous coupling strengths may destabilize cluster solutions or give rise to new stable cluster solutions.

**Figure 9:**
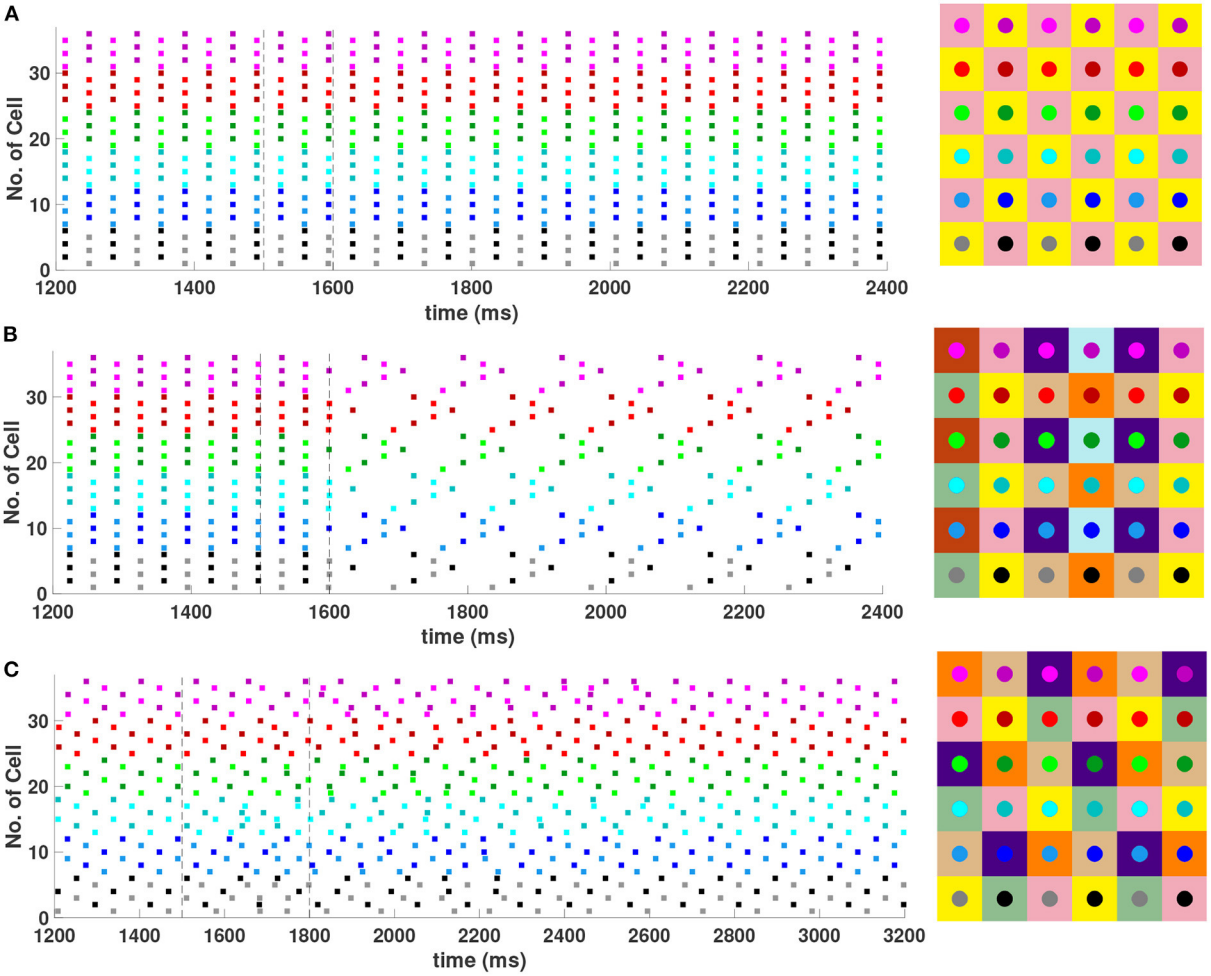
Stable cluster solutions in the 6 × 6 network. Cells are coupled to their first nearest neighbors in the horizontal, vertical, and diagonal directions, and to their second nearest neighbors in the horizontal and vertical directions. **(A,B)** The effect of heterogeneous coupling strengths on the stability of the 2-cluster diagonal stripe solution; **(A)** With homogeneous coupling strengths (*h*_1_ = *v*_1_ = *d* = *h*_2_ = *v*_2_ = 1) the network returns to the 2-cluster diagonal stripe solution after a sufficiently small perturbation is applied for *t*∈[1500, 1600] ms. **(B)** With stronger coupling to the first nearest neighbors in the diagonal direction and to the second nearest neighbors in horizontal and vertical directions (*h*_1_ = *v*_1_ = 1, *d* = *h*_2_ = *v*_2_ = 4), the same perturbation switches the network to a stable 8-cluster solution. **(C)** Stability of (3, 6) 6-cluster solution with weaker coupling to the first nearest neighbors in the vertical direction (*h*_1_ = *d* = *h*_2_ = *v*_2_ = 1, *v*_1_ = 0.4). The network starts on the solution and then returns to it after a transient perturbation applied for *t*∈[1500, 1800] ms. The corresponding 2D clusters for each stable solution after transient perturbations are shown next to each raster plot.

It was shown in Section 4.1 that with first nearest neighbor coupling only (*h*_1_ = *v*_1_ = *d, h*_2_ = *v*_2_ = 0), the 2-cluster diagonal stripe solution in the 6 × 6 network is stable, but with sufficiently strong diagonal coupling (*d* > ≈ 7.59*h*_1_) this solution can be destabilized. See Equation (18). Numerical simulations of the network (not shown) confirm that with *h*_1_ = *v*_1_ = 1, *h*_2_ = *v*_2_ = 0, a value of *d* > 7.6 is required to destabilize this solution. Further evaluation of the eigenvalues given by Equation (15) for the 6 × 6 network shows that sufficiently strong diagonal and second nearest neighbor coupling (*d* = *h*_2_ = *v*_2_>*h*_1_ = *v*_1_) should also be able to destabilize the 2-cluster diagonal stripe solution. Numerical simulations of the Wang-Buzsaki inhibitory network confirming this are shown in Figure 9. In Figure 9A with homogeneous coupling strengths (*h*_1_ = *v*_1_ = *d* = *h*_2_ = *v*_2_ = 1), the network is initialized on the 2-cluster diagonal stripe solution and then returns to this solution after a transient perturbation to a certain subset of cells is applied at *t* ∈ [1500, 1600] in the network. This demonstrates the stability of this solution. In Figure 9B, heterogeneous coupling with stronger second nearest neighbor and diagonal coupling is introduced (*h*_1_ = *v*_1_ = 1, *d* = *h*_2_ = *v*_2_ = 4). Here, the same perturbation applied to the 2-cluster diagonal solution switches the network to a stable 8-cluster solution.

With homogeneous coupling strengths in a 6 × 6 network, (*p*_*h*_, *p*_*v*_) 6-cluster solutions with *p*_*v*_ = 6 are unstable (Section 4.1). However, if the horizontal coupling to the first nearest neighbors becomes stronger than the vertical coupling (e.g., *h*_1_ = *d* = *h*_2_ = *v*_2_ = 1 but *v*_1_ = 0.4), this heterogeneity gives rise to a stable (3, 6) 6-cluster solution such that $\psi_{h}=\frac{2\pi }{3}$ and $\psi_{v}=\frac{\pi }{3}$ (Figure 9C). In this solution, cells across rows break into 3 clusters while cells along columns separate into 6 clusters, as illustrated in Figure 2H.

We can further break the coupling symmetry in our networks if we relax the assumption that the connectivity matrix *W* is symmetric, that is, if we let *w*_*i, j*_ ≠ *w*_*j, i*_. This returns us to the general stability condition (12). Note that this condition depends on *H*, not *H*_*odd*_ and, from Figures 3B,D, there is asymmetry in *H*(ϕ). This could lead to interesting results if *W* is not symmetric.

## 5. Discussion

We have shown the existence of synchronized cluster solutions and found their stability conditions in networks of intrinsically oscillating neurons on a general 2D torus. The stability conditions are derived generally for potential coupling to nearest neighbors in the vertical and horizontal directions, nearest neighbors in the diagonal directions, and second nearest neighbors in the vertical and horizontal directions. We considered specific solutions arising from various connectivity configurations and different coupling strengths. We also considered heterogeneous coupling strengths. We showed that cluster solutions could change stability if we add or remove connections between nearest neighbors in the diagonal directions or second nearest neighbors in the vertical and horizontal directions. We then used simulations of biophysical inhibitory neural networks (4 × 4 and 6 × 6) to verify our analytical predictions about which solutions would be stable under various configurations. See Tables 2, 3 for a summary of the results with homogeneous coupling. For some solutions, the clusters in the 2D network can be thought of as generalizations of cluster solutions that occur in 1D networks, where isolating a ring in the horizontal or vertical direction results in a solution with constant phase differences between neighboring cells. Examples of this would be the diagonal, horizontal, and vertical stripe solutions. However, the 2D nature of the coupling introduces other solutions that are not simple generalizations of 1D solutions. For example we found *p*-cluster solutions with *p*_*h*_ clusters in the horizontal direction and *p*_*v*_ clusters in the vertical direction. Further, in our numerical simulations we observed stable solutions where the phase differences between neighboring cells are not uniform across the network but do follow a regular pattern, such as in Figures 5B, 8A, and stable solutions where the number of neurons in different clusters is different, such as in Figure 9B. While the stability of some of these observed solutions makes sense intuitively, we have not yet been able to find general stability conditions for these types of solution.

**Table 2:** Cluster solutions for the 4 × 4 network and the stability as predicted by the phase model analysis for various homogeneous coupling arrangements.

**Clusters**	**Type**	**ψ_*h*_**	**ψ_*v*_**	***h*_1_ = *v*_1_>0**	***h*_1_ = *v*_1_ = *d*>0**
	Horizontal	0	π	Unstable*	Stable*
2	Vertical	π	0	Unstable	Stable*
	Diagonal	π	π	Stable*	Stable*
	Horizontal	0	$\frac{\pi }{2},\frac{3\pi }{2}$	Unstable	Unstable
4	Vertical	$\frac{\pi }{2},\frac{3\pi }{2}$	0	Unstable	Unstable
	Diagonal	$\frac{\pi }{2},\frac{3\pi }{2}$	$\frac{\pi }{2},\frac{3\pi }{2}$	Unstable	Unstable
	Diagonal	$\frac{\pi }{2},\frac{3\pi }{2}$	$\frac{3\pi }{2},\frac{\pi }{2}$	Unstable	Unstable
	(2, 4)	π	$\frac{\pi }{2},\frac{3\pi }{2}$	Unstable	Unstable
	(4, 2)	$\frac{\pi }{2},\frac{3\pi }{2}$	π	Unstable	Unstable

Solutions marked with a * have been verified numerically.

**Table 3:** Cluster solutions for the 6 × 6 network and the stability as predicted by the phase model analysis for various homogeneous coupling arrangements.

**Clusters**	**Type**	**ψ_*h*_**	**ψ_*v*_**	***h*_1_ = *v*_1_>0**	***h*_1_ = *v*_1_ = *d*>0**	***h*_1, 2_ = *v*_1, 2_ = *d*>0**
2	Horizontal	0	π	Unstable	Stable*	Stable*
	Vertical	0	π	Unstable	Stable	Stable
	Diagonal	π	π	Stable*	Stable	Stable
3	Horizontal	0	$\frac{2\pi }{3},\frac{4\pi }{3}$	Unstable	Stable*	Stable
	Vertical	$\frac{2\pi }{3},\frac{4\pi }{3}$	0	Unstable	Stable*	Stable*
						
	Diagonal	$\frac{2\pi }{3},\frac{4\pi }{3}$	$\frac{2\pi }{3},\frac{4\pi }{3}$	Stable	Stable	Stable
	Diagonal	$\frac{2\pi }{3},\frac{4\pi }{3}$	$\frac{4\pi }{3},\frac{2\pi }{3}$	Stable*	Stable	Stable
6	Horizontal	0	$\frac{\pi }{3},\frac{5\pi }{3}$	Unstable	Unstable	Unstable
	Vertical	$\frac{\pi }{3},\frac{5\pi }{3}$	0	Unstable	Unstable	Unstable
	Diagonal	$\frac{\pi }{3},\frac{5\pi }{3}$	$\frac{\pi }{3},\frac{5\pi }{3}$	Unstable	Unstable	Unstable
	Diagonal	$\frac{\pi }{3},\frac{5\pi }{3}$	$\frac{5\pi }{3},\frac{\pi }{3}$	Unstable	Unstable	Unstable
						
	(2, 3)	π	$\frac{2\pi }{3},\frac{4\pi }{3}$	Stable*	Unstable	Unstable
	(3, 2)	$\frac{2\pi }{3},\frac{4\pi }{3}$	π	Stable	Unstable	Unstable
	(2, 6)	π	$\frac{\pi }{3},\frac{5\pi }{3}$	Unstable	Stable*	Stable
	(6, 2)	$\frac{\pi }{3},\frac{5\pi }{3}$	π	Unstable	Stable	Stable
	(3, 6)	$\frac{2\pi }{3},\frac{4\pi }{3}$	$\frac{\pi }{3},\frac{5\pi }{3}$	Unstable	Unstable	Unstable
	(6, 3)	$\frac{\pi }{3},\frac{5\pi }{3}$	$\frac{2\pi }{3},\frac{4\pi }{3}$	Unstable	Unstable	Unstable

Solutions marked with a * have been verified numerically.

Our existence and stability analyses apply to general *m*×*n* 2D networks. While our numerical simulations focused on square (*m* = *n*) 2D networks, similar types of solutions will be exhibited in rectangular (*m*≠*n*) as long as the existence and stability conditions are met. For example, 2-cluster horizontal (vertical) stripe solutions will exist in any size network that has an even number of rows (columns). Similarly, 2-cluster (3-cluster) diagonal stripe solutions will exist in any size network where the number of rows and columns is even (divisible by 3). Additionally, the types of (*p*_*h*_, *p*_*v*_) *p*-cluster solutions that we observed in square networks will exist in rectangular networks as long as the number of columns is divisible by *p*_*h*_ and the number of rows is divisible by *p*_*v*_. The stability of any of these solutions can be determined using our expressions for the eigenvalues.

Diagonal coupling seems to be particularly important in determining the stable cluster solutions and differentiates the 2D network from the 1D network. Without diagonal coupling, if either the *p*_*h*_- or *p*_*v*_-cluster solution is unstable for the 1D network, then the (*p*_*h*_, *p*_*v*_) *p*-cluster solution is unstable for the 2D network. However, adding diagonal coupling increases the number of predicted stable solutions. For example, in the 6 × 6 network, the number of stable solutions increases from 9 to 15 (see Table 3). We can also find new types of *p*-cluster solutions for the 2D network. Even if *p*-cluster solutions cannot exist in 1D networks with *n* and *m* neurons, there can exist *p*-cluster solutions in the 2D *m*×*n* networks if there are *p*_*h*_, *p*_*v*_ (which divide *m, n*, respectively) such that *p* = lcm(*p*_*h*_, *p*_*v*_).

While our analysis results can be applied to any type of network, we chose to focus on networks of inhibitory neurons. Intuitively, one might expect that the most stable configurations in such networks would be when neurons that are connected don't fire synchronously, equivalently, that the clusters would contain neurons that are not directly coupled. Indeed we found a very stable 4-cluster solution in both the 4 × 4 and 6 × 6 networks where neurons in the same cluster were minimally connected (see Figures 5B, 8A). However, we also found stable solutions where neurons in the same cluster are directly coupled. The stability of many of these solutions was enabled by the 2D structure of the network and specifically the diagonal coupling discussed above. The relative phases of neurons in different clusters is an important factor in determining stability. In phase model analysis the effect of the relative phase on stability is determined by the slope of the interaction function, at the relative phase (see Figure 3). Not surprisingly, we found that solutions with relative phases of π are the most strongly stable; however solutions with any relative phases $\phi \in \left[\frac{17\pi }{32},\pi \right]$ are also stable. Interestingly, the most strongly destabilizing relative phase was not 0, but around $\frac{3\pi }{16}$. This makes sense if we keep in mind that coupling between cells is not instantaneous in our model network but through simulated chemical synapses which introduce a small delay. Such a small delay changes the phase when the inhibition is felt by the post-synaptic neuron. Stability of a solution in a network with a particular coupling structure occurs when the coupling strength is stronger between neurons with relative phases that are stabilizing than between neurons whose relative phases are destabilizing.

Cluster solutions in model networks have been linked to neural assemblies in the brain as these are solutions where the neurons form groups, with neurons in the same group firing synchronously and neurons in different groups firing phase-locked with non-zero phase difference. Our analysis results show how such assemblies can arise spontaneously, generated only by the connectivity of the network and the dynamics of the neurons. In our model of 2D networks of intrinsically oscillatory neurons, a vast range of cluster solutions can exist, with multiple stable solutions occurring for the same parameter values. For the small 6 × 6 inhibitory networks we studied in detail, with certain coupling configurations we found four different stable cluster solutions in our numerical simulations. Larger networks would be expected to have more such solutions. In these simulations, we were able to switch the network between solutions by adding transient input to a subset of the neurons. This gives one mechanism for the formation of neural assemblies, where the different stable cluster solutions represent different assemblies and transient external input to the network switches the system from one assembly to another. We note that the number of clusters in the solution affects the network level behavior. For example, a 2-cluster solution will give a network firing rate that is twice the intrinsic firing rate of the neurons. Thus, the co-existence of stable cluster solutions gives a mechanism for the network to respond to different transient inputs with different network firing rates. We also showed how changing the coupling configuration could switch the stable cluster solutions that occur. For example, adding diagonal coupling could increase the number of stable solutions and changing the relative strengths of different coupling connections could change the stability of cluster solutions. Thus, changes in network connectivity gives another mechanism for changing the number and composition of the neural assemblies that a network can exhibit. In our example simulations, neurons formed connections with between 4 and 12 of their neighbors. Thus, the connectivity ranged from sparse (~10% for 6 × 6 networks with 4 connections) to dense (~75% connectivity for 4 × 4 networks with 12 connections). Our results indicate that the structure of connectivity is more important for forming clusters than density of connectivity.

Neural assembly firing has been identified in the striatum, a sparsely coupled, inhibitory network that is part of the basal ganglia circuit (Carrillo-Reid et al., 2008; Miller et al., 2008; Adler et al., 2013; Barbera et al., 2016; Klaus et al., 2017). Based on an extensive review of anatomical and experimental evidence, Burke et al. (2017) proposed that lateral inhibition between spiny neurons in the striatum organizes the cells into assemblies, with different assemblies corresponding to different behaviors. Our work supports this proposal. In our model, the external input that switches between assemblies would correspond to excitatory input to the striatum from the cortex or thalamus. Further, the strength of synapses in the striatum can be affected by changes in dopamine level (Lemos et al., 2016; Dobbs et al., 2017), which would correspond to changing the connections strengths in our network. Of course our model is much simpler than the striatum, which includes multiple different populations of spiny neurons as well as several populations of interneurons (Burke et al., 2017). Future work could include extending our model to include more populations.

Recent work on memory formation suggests that some brain regions have pre-configured neural ensembles, allowing memories to be encoded quickly (Grosmark and Buzsáki, 2016; Farooq et al., 2019; Miyawaki and Mizuseki, 2022). The internal generation of multiple assemblies we find in our study gives a mechanism for this. Although the model networks in our simulations were inhibitory, the analysis does not depend on the type of coupling. A topic for future work would be to apply our results to excitatory or excitatory/inhibitory networks.

It would be interesting to see how our work extends to more biologically realistic coupling regimes. One obvious extension would be to take the continuum limit (i.e., *m, n* → ∞), resulting in a 2D partial differential equation for the phases of the neurons, ϕ(*x, y*). While there have been several studies of 1D continuum models (Ermentrout, 1992; Strogatz, 2000; Wiley et al., 2006; Kazanci and Ermentrout, 2007; Sethia et al., 2011; Girnyk et al., 2012; Omel'chenko et al., 2014; Heitmann and Ermentrout, 2015), we know of only one study of a 2D continuum model (Heitmann et al., 2012). Analysis of the existence and stability of wave solutions (the continuum analog of our phase-locked cluster solutions) has only been done in 1D models. This work has primarily focused on either homogeneous coupling or symmetric distance-dependent coupling, and either excitatory or center-surround coupling, where neurons receive excitation from near neighbors and inhibition from further neighbors.The extension to purely inhibitory 2D networks can in principle be done, although the analysis may be challenging. Our work on discrete networks and numerical studies of discrete and continuum models (Heitmann et al., 2012; Spreizer et al., 2017) indicates that it would be of particular interest to consider non-monotonic coupling and heterogeneous coupling. Further, some interesting behavior in our models results from the non-square matrices, which could be incorporated by choosing different length scales in a continuum model. Another extension of our work would be to relax or change the structure in the connectivity matrix. Other biologically relevant possibilities include small-world networks and networks with hubs (Bullmore and Sporns, 2009; Bassett and Bullmore, 2017).

## Data availability statement

The datasets presented in this study can be found in online repositories. The name of the repository and accession number can be found below: ModelDB, https://modeldb.yale.edu/267329.

## Author contributions

SC, JM, and XW conducted the phase model analyses. VB and HR implemented and conducted numerical simulations. All authors contributed to conceptualization, methodology, writing the original draft, reviewing and editing the final manuscript. All authors contributed to the article and approved the submitted version.

## Funding

All authors received support from the American Institute of Mathematics Structured Quartet Research Ensembles program. SC was supported by the Natural Sciences and Engineering Research Council of Canada.

## Conflict of interest

The authors declare that the research was conducted in the absence of any commercial or financial relationships that could be construed as a potential conflict of interest.

## Publisher's note

All claims expressed in this article are solely those of the authors and do not necessarily represent those of their affiliated organizations, or those of the publisher, the editors and the reviewers. Any product that may be evaluated in this article, or claim that may be made by its manufacturer, is not guaranteed or endorsed by the publisher.
